# Unstable Mechanisms of Resistance to Inhibitors of Escherichia coli Lipoprotein Signal Peptidase

**DOI:** 10.1128/mBio.02018-20

**Published:** 2020-09-08

**Authors:** Homer Pantua, Elizabeth Skippington, Marie-Gabrielle Braun, Cameron L. Noland, Jingyu Diao, Yutian Peng, Susan L. Gloor, Donghong Yan, Jing Kang, Anand Kumar Katakam, Janina Reeder, Georgette M. Castanedo, Keira Garland, Laszlo Komuves, Meredith Sagolla, Cary D. Austin, Jeremy Murray, Yiming Xu, Zora Modrusan, Min Xu, Emily J. Hanan, Sharookh B. Kapadia

**Affiliations:** a Department of Infectious Diseases, Genentech, South San Francisco, California, USA; b Department of OMNI Bioinformatics, Genentech, South San Francisco, California, USA; c Department of Chemistry, Genentech, South San Francisco, California, USA; d Department of Structural Biology, Genentech, South San Francisco, California, USA; e Department of Biochemical and Cellular Pharmacology, Genentech, South San Francisco, California, USA; f Department of Translational Immunology, Genentech, South San Francisco, California, USA; g Department of Pathology, Genentech, South San Francisco, California, USA; h Department of Molecular Biology, Genentech, South San Francisco, California, USA; McMaster University

**Keywords:** heteroresistance, lipoprotein, Lpp, LspA, globomycin

## Abstract

Clinical development of antibiotics with novel mechanisms of action to kill pathogenic bacteria is challenging, in part, due to the inevitable emergence of resistance. A phenomenon of potential clinical importance that is broadly overlooked in preclinical development is heteroresistance, an often-unstable phenotype in which subpopulations of bacterial cells show decreased antibiotic susceptibility relative to the dominant population. Here, we describe a new globomycin analog, G0790, with potent activity against the Escherichia coli type II signal peptidase LspA and uncover two novel resistance mechanisms to G0790 in the clinical uropathogenic E. coli strain CFT073. Building on the previous finding that complete deletion of Lpp, the major Gram-negative outer membrane lipoprotein, leads to globomycin resistance, we also find that an unexpectedly modest decrease in Lpp levels mediated by insertion-based disruption of regulatory elements is sufficient to confer G0790 resistance and increase sensitivity to serum killing. In addition, we describe a heteroresistance phenotype mediated by genomic amplifications of *lspA* that result in increased LspA levels sufficient to overcome inhibition by G0790 in culture. These genomic amplifications are highly unstable and are lost after as few as two subcultures in the absence of G0790, which places amplification-containing resistant strains at high risk of being misclassified as susceptible by routine antimicrobial susceptibility testing. In summary, our study uncovers two vastly different mechanisms of resistance to LspA inhibitors in E. coli and emphasizes the importance of considering the potential impact of unstable and heterogenous phenotypes when developing antibiotics for clinical use.

## Abstract (precis)

Despite increasing evidence suggesting that antibiotic heteroresistance can lead to treatment failure, the significance of this phenomena in the clinic is not well understood, because many clinical antibiotic susceptibility testing approaches lack the resolution needed to reliably classify heteroresistant strains. Here we present G0790, a new globomycin analog and potent inhibitor of the Escherichia coli type II signal peptidase LspA. We demonstrate that in addition to previously known mechanisms of resistance to LspA inhibitors, unstable genomic amplifications containing *lspA* can lead to modest yet biologically significant increases in LspA protein levels that confer a heteroresistance phenotype.

## INTRODUCTION

Antibiotic resistance can be generated via multiple mechanisms, including target alteration, modification/degradation of the antibiotic molecule, and decreased cell penetration or enhanced efflux (reviewed in reference 1). In some cases, seemingly isogenic bacteria can exhibit phenotypic heterogeneity with respect to antibiotic resistance and can grow in the presence of antibiotic therapy, a phenomenon referred to as heteroresistance (2). Heteroresistance has been observed in a range of Gram-positive (3–5) and Gram-negative (6–9) bacterial pathogens and can be mediated by mutations (single nucleotide polymorphisms [SNPs], insertions, or deletions) or tandem amplifications (10). Heteroresistance is usually unstable and can be due to either the loss of the modification or a gain of secondary mutations elsewhere in the genome, ultimately leading to loss of the resistance phenotype in the absence of selective antibiotic pressure. While heteroresistance has been studied for more than 50 years, there is increasing evidence of its presence in clinical isolates (10–12). Many clinical laboratories use nonselective culture techniques to grow bacterial isolates from patient samples (13), and there are currently no established antimicrobial susceptibility tests to identify heteroresistance in the hospital setting. For these reasons, heteroresistance phenotypes can escape detection and potentially pose a significant hurdle to the appropriate administration of antibiotics to patients. Given there is an urgent clinical need for new antibiotics with novel mechanisms of action to combat the rise of infections caused by multidrug-resistant pathogens, heteroresistance to preclinical candidates must be assessed.

Bacterial lipoprotein biosynthesis is an attractive target for novel antibiotic drug discovery, as bacterial lipoproteins play critical roles in adhesion, nutrient uptake, antibiotic resistance, virulence, invasion, and immune evasion (14). Lipoprotein biosynthesis in Gram-negative bacteria is mediated by three essential inner membrane-localized enzymes (14), which work sequentially to generate the mature triacylated lipoproteins. The second enzyme in the pathway, prolipoprotein signal peptidase (LspA), is an aspartyl endopeptidase which cleaves off the signal peptide N-terminal to a conserved diacylated +1 cysteine, diacylated by the first enzyme in the cascade, Lgt (15). While essential for growth of most Gram-negative bacteria, *lspA* is not essential for *in vitro* growth of Gram-positive bacteria but does lead to attenuation in virulence (16–18). LspA is the target of the natural product antibiotics globomycin (GBM) and myxovirescin (TA) synthesized by *Streptomyces* species and Myxococcus xanthus, respectively (19, 20), which inhibit LspA function by targeting the catalytic dyad aspartic acid residues (21). Escherichia coli harbors >90 lipoproteins, many of which are localized to the inner leaflet of the outer membrane but can also be exposed on the bacterial cell surface (22, 23). One of the main outer membrane lipoproteins is a small ∼8-kDa lipoprotein called Lpp (or Braun’s lipoprotein) (24). Lpp has been demonstrated to be critical for maintaining membrane integrity and permeability mediated through a covalent linkage between the ε-amino group of the C-terminal lysine residue in Lpp and the *meso*-diaminopimelic acid residue on the peptidoglycan peptide stem (25–27). E. coli mutants deficient in Lpp exhibit increased outer membrane (OM) permeability, leakage of periplasmic components, and increased outer membrane vesicle (OMV) release (28, 29). LspA inhibitors are proposed to cause bacterial cell death by leading to the accumulation of the peptidoglycan-linked form of Lpp in the inner membrane. Consistent with this hypothesis, deletion of *lpp* leads to resistance to certain inhibitors of lipoprotein biosynthesis and transport (30–33). However, all these studies were performed using the laboratory-adapted E. coli MG1655 K-12 strain, and a more rigorous understanding of the complete resistance profile in a clinical isolate is warranted.

Given the challenges associated with identifying new antibiotic leads that can efficiently penetrate the asymmetric Gram-negative bacterial cell envelope from initial hits identified in high-throughput screens (34, 35), we decided to use the natural product GBM as a starting point for medicinal chemistry optimization using published and in-house cocrystal structures to guide design of GBM analogs. This effort was published separately and culminated in the discovery of several advanced LspA inhibitors, including G0790 (36). In this study, we further explore the activity of G0790, a GBM analog with significantly increased potency against multiple members of the *Enterobacteriaceae* family, including *Escherichia*, *Enterobacter*, and *Klebsiella* species. As antibacterial molecules that inhibit single protein targets are expected to be more susceptible to resistance emergence than those which inhibit multicomponent molecular structures (37), we sought to initially understand the resistance profile to G0790 using the clinical uropathogenic E. coli strain CFT073. We demonstrate that in addition to the previously described resistance mechanism of *lpp* deletion, even a modest downregulation of the major Gram-negative bacterial outer membrane lipoprotein Lpp confers resistance to G0790. We also identify a novel heteroresistance phenotype mediated by unstable genomic amplifications of *lspA* leading to modestly increased LspA protein levels in the inner membrane and a concomitant increase in the MIC. Our results add to an increasing appreciation of the potential impact of heteroresistance on the administration of appropriate antibiotic therapy and suggest that studies to assess heteroresistance should be performed during preclinical development of novel antibacterial candidates.

## RESULTS

### G0790 is a potent inhibitor of E. coli LspA.

**FIG 1:**
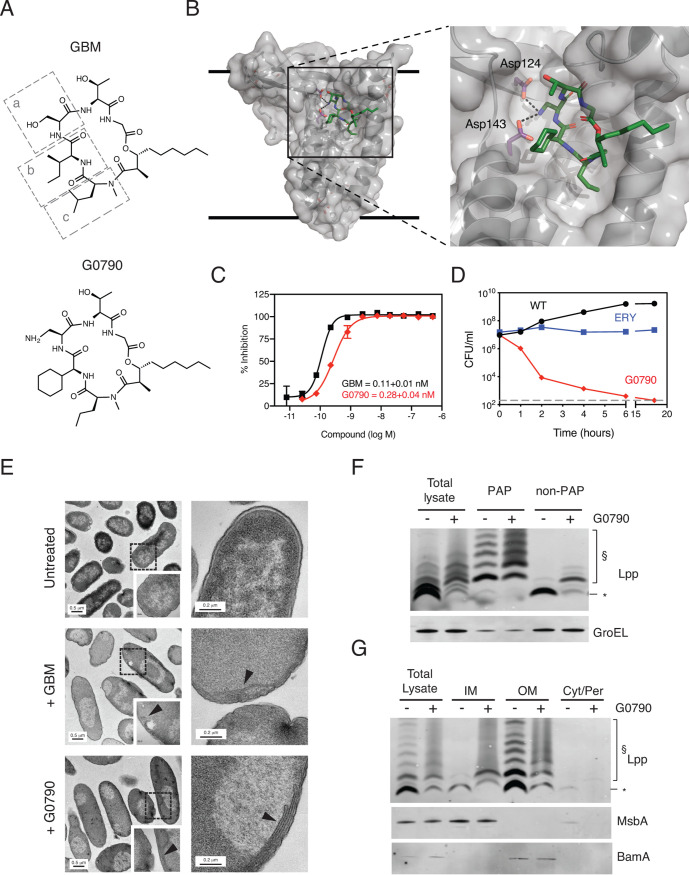
Identification of G0790 as a potent globomycin analog. (A) Chemical structures of globomycin (GBM) and G0790 showing modified side chains at positions “a” [serine to (*S*)-2,3-diaminopropionic acid], “b” (*allo*-isoleucine to cyclohexylglycine), and “c” (*N*-methyl-leucine to *N*-methyl-norvaline). (B) A molecular model of G0790 bound to LspA was built based on the crystal structure of the LspA in complex with globomycin (PDB ID 5DIR). The model reveals that the (*S*)-2,3-diaminopropionic acid engages the two catalytic aspartate residues (D124 and D143, magenta), while the cyclohexylglycine and *N-*methyl-norvaline are easily accommodated in the transmembrane region. The thick black lines represent the predicted boundaries of the lipid bilayer, G0790 is shown as green sticks with the molecular surface of LspA rendered as a transparent surface and the secondary structure show as cartoons. (C) Dose-dependent inhibition of LspA biochemical activity by GBM (black) or G0790 (red). Inhibitors were incubated with LspA and the diacylated E. coli Pal peptide substrate, and cleavage of the signal peptide was monitored by mass spectrometry as discussed in Materials and Methods. Inhibition of LspA activity is graphed (means ± SDs) as percent inhibition normalized to reactions performed in the absence of any inhibitors. Data are representative of two independent experiments each performed in triplicates. (D) G0790 is bactericidal. E. coli CFT073 was left untreated (black) or treated with 2× MICs of G0790 (red) or erythromycin (ERY; blue), and CFU were enumerated at various times posttreatment. These data are representative of two independent experiments, each performed in duplicates. (E) G0790 leads to changes in cellular morphology and membrane accumulation in CFT073. CFT073 was treated with 2× MICs of GBM or G0790 for 1 h and processed for visualization by electron microscopy. Arrowheads denote accumulation of bacterial cell membranes. (F and G) G0790 treatment of CFT073 cells leads to loss of the peptidoglycan-associated form of Lpp in the outer membrane and its accumulation in the inner membrane. CFT073 was treated with 2× MIC G0790, and Lpp expression was detected by Western blotting in peptidoglycan-associated protein fractions (PAP) (F) or inner versus outer membrane fractions (G). *, mature non-peptidoglycan-associated Lpp; §, peptidoglycan-associated Lpp forms. GroEL was used as a loading control for total lysates as well as to monitor enrichment of PAP versus the nonpeptidoglycan protein fraction (non-PAP). As controls for the membrane isolation, inner and outer membrane fractions were probed with antibodies against an inner (MsbA) and outer (BamA) membrane protein. These data are representative of at least four independent experiments.

GBM, a natural product LspA inhibitor, is not a suitable clinical antibacterial candidate due to inefficient penetration through the impermeable Gram-negative bacterial outer membrane, resulting in weak growth inhibition against wild-type (WT) Gram-negative bacteria (38). Therefore, with the goal of increasing GBM whole-cell potency through medicinal chemistry optimization, we identified G0790 as an analog with increased growth inhibitory activity against multiple Gram-negative bacterial species (36). G0790 contains (*S*)-2,3-diaminopropionic acid (Dap), cyclohexylglycine (Chg), and *N*-methyl-norvaline (Nva) residues in place of the serine, *allo*-isoleucine, and *N*-methyl-leucine amino acids at positions a, b, and c, respectively, of the GBM molecule (Fig. 1A). Molecular modeling based on the crystal structure of the LspA in complex with globomycin (PDB identifier [ID] 5DIR) suggests that Dap engages the two catalytic aspartate residues (D124 and D143) (Fig. 1B). The MIC, or amount of compound required to completely inhibit bacterial cell growth, for G0790 was 4- to 8-fold lower than for the parent GBM against WT E. coli (CFT073), Enterobacter cloacae (ATCC 13047), and Klebsiella pneumoniae (ATCC 700603) strains (Table 1). To determine if the increased WT E. coli activity was driven by decreased efflux, we tested G0790 in a deletion strain (E. coli MG1655 Δ*tolC*), which lacks the outer membrane protein TolC component of major E. coli efflux pump AcrAB-TolC (39). The WT/*tolC* MIC shifts for GBM and G0790 were ∼128-fold and ∼31-fold, respectively, suggesting reduced efflux contributes to the improved WT E. coli activity (Table 1). A modestly decreased MIC against Acinetobacter baumannii (ATCC 17978) was also observed compared to that for GBM (Table 1). Given the sequence differences between LspA homologs from E. coli and A. baumannii, we wanted to confirm whether A. baumannii LspA was sensitive to GBM. The *lspA* homologs from two A. baumannii strains (ATCC 17978 and ATCC 19606) rescued growth of an E. coli *lspA* inducible deletion strain (MG1655 Δ*lspA*) (see Fig. S1A in the supplemental material), consistent with the high conservation of the LspA active site residues across multiple bacterial genera (40). G0790 and GBM equivalently inhibited LspA enzymatic activity to similar extents *in vitro* (0.28 ± 0.04 nM and 0.11 ± 0.01 nM, respectively) (Fig. 1C). Overall, our data suggest that G0790 has gained whole-cell potency against multiple clinically relevant Gram-negative bacterial species.

**TABLE 1:** MICs of G0790 against a panel of Gram-negative bacterial species

Strain	MIC (mg/liter) (*n* = 2)	
	Globomycin	G0790
E. coli CFT073	32	4
E. coli CFT073 Δ*lpp*	128	16
E. coli CFT073(*pBADlspA*)	>128	64
E. coli CFT073 plus 50% HS^*a*^	128	24
E. coli CFT073 *imp4213*	0.06	0.02
E. coli CFT073 *imp4213* Δ*lpp*	4	1
E. coli CFT073 *imp4213*(*pBADlspA*)	16	4
E. coli *MG1655*	32	4
E. coli *MG1655* Δ*tolC*	0.25	0.13
E. cloacae 13047	64	8
K. pneumoniae 700603	64	16
A. baumannii 17978	128	64
S. aureus USA300	>128	>128

a HS, human serum.

10.1128/mBio.02018-20.1 FIG S1 (A) Growth of an E. coli strain lacking *lspA* can be rescued by complementing with *lspA* from A. baumannii. WT E. coli MG1655 (black squares), MG1655 *lspA* inducible deletion strain containing an empty pLMG18 plasmid (MG1655 Δ*lspA*; red circlea) or pLMG18 expressing *lspA* from E. coli (MG1655 Δ*lspA*:*lspA^Ec^*, blue triangles), A. baumannii 17978 (MG1655 Δ*lspA*:*lspA^Ab17978^*; yellow diamonds), or A. baumannii 19606 (MG1655 Δ*lspA*:*lspA^Ab19606^*; green diamonds) were grown in the presence of 0.2% glucose, and CFU were enumerated over time. The gray dashed line represents the limit of detection (200 CFU/ml) for the experiment. Data are representative of two independent experiments each performed in quadruples. (B) Live-cell imaging of untreated WT CFT073 or CFT073 treated with G0790. The CFT073 cells contain a plasmid that expresses GFP in the cytoplasm. Fluorescence images were overlaid with the phase contrast images. Bar, 2 μm. Download FIG S1, PDF file, 1.6 MB. Copyright © 2020 Pantua et al. 2020 Pantua et al. This content is distributed under the terms of the Creative Commons Attribution 4.0 International license.

We confirmed that the increase in potency against WT bacterial strains was specific to LspA by using multiple parallel approaches. First, inhibition of WT CFT073 and a mutant of CFT073 that exhibits increased outer membrane permeability (CFT073 *imp4213*) by G0790 was decreased upon LspA overexpression or *lpp* deletion, similar to what has been previously described for GBM (Table 1). Second, G0790 shows no increased activity against the Gram-positive bacteria Staphylococcus aureus USA300 in which *lspA* is known to be dispensable for *in vitro* growth (41). Third, like GBM, G0790 is bactericidal, and treatment with G0790 leads to a globular cell morphology and accumulation of bacterial membranes in CFT073 cells (Fig. 1D and E and Fig. S1B). Finally, consistent with previous results using GBM (42), G0790 treatment of CFT073 leads to an accumulation of the peptidoglycan-associated form of Lpp in the inner membrane (Fig. 1F and G). These data confirm that potent analog G0790 maintains LspA-specific growth inhibitory activity against WT bacteria, thereby allowing us to perform a comprehensive assessment of G0790 resistance mechanisms in a clinical E. coli isolate.

### Lpp downregulation is sufficient to lead to G0790 resistance.

**FIG 2:**
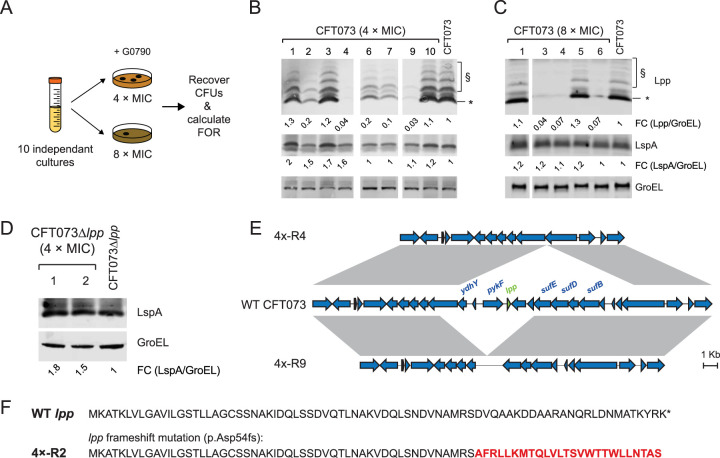
Selection of G0790-resistant CFT073. (A) Schematic representing the strategy to select for G0790-resistant mutants. Ten independent overnight cultures of E. coli CFT073, CFT073 Δ*lpp*, or CFT073 *imp4213* were spread on cation-adjusted MHII agarose plates containing G0790 at 2× and 4× the MIC. For E. cloacae 13047 and K. pneumoniae 700603, we tested three independent overnight cultures due to compound limitations. Resistance frequencies were calculated by dividing the number of colonies formed after a 48-h incubation at 37°C by the total CFU count initially spread on the plates (Table 2). ND, not done. Expression of Lpp and LspA in G0790-resistant CFT073 cells growing on MHII agarose plates containing 4× MIC (B) or 8× MIC (C) G0790 concentrations or G0790-resistant CFT073 Δ*lpp* cells growing on MHII agarose plates containing 4× MIC G0790 concentrations (D). Relative expression levels of Lpp and LspA were calculated by the ratio to GroEL and normalized to WT CFT073 or CFT073 Δ*lpp* (which was set at 1). Of note, CFT073 4×-R5, 4×-R8, and 8×-R2 were determined to be mixed colonies by WGS and hence were not followed up in the remainder of this study. (E) Gene maps for two representative G0790-resistant strains containing genomic deletions encompassing *lpp* (4×-R4 and 4×-R9) compared to the wild type. Pairwise BLAST identities are indicated by gray shading and show conserved regions among the strains. (F) Putative sequence of mutant Lpp protein encoded by CFT073 4×-R2 containing the Asp54 frameshift mutation (p.Asp43fs).

To identify the mechanisms of G0790 resistance in E. coli CFT073, we performed frequency of resistance (FOR) studies by plating WT CFT073 cells on Mueller-Hinton II (MHII) agarose plates containing G0790 at 4× or 8× the MIC. The FOR was determined by calculating the ratio of CFU that grew out on the G0790-containing MHII agarose plates to the initial input CFU (Fig. 2A). We used CFT073 *imp4213* instead of WT CFT073 when determining FOR to GBM given its weak activity against WT bacteria. Low FORs to G0790 were detected in WT CFT073 (2.9 × 10^−9^ and 6.8 × 10^−11^ selected at 4× and 8× MIC, respectively), similar to those measured for GBM in CFT073 *imp4213* (Table 2). An ∼85-fold decrease in G0790 FORs was observed in CFT073 cells with *lpp* deleted (CFT073 Δ*lpp*) compared to that in WT CFT073 (Table 2). While G0790 FORs selected at 4× MICs in E. cloacae 13047 were similar to those determined with E. coli, FORs in K. pneumoniae 700603 were ∼17-fold higher (Table 2). These data now allow us to fully profile the resistance mechanisms to LspA inhibitors in a WT E. coli clinical isolate.

**TABLE 2:** Frequency of resistance for globomycin and G0790 against *Enterobacteriaceae* species

Bacterial strain	LspA inhibitor		CFU (per plate)										Total CFU plated	FOR^*a*^	*P* value^*b*^
	Name	Concn	P1	P2	P3	P4	P5	P6	P7	P8	P9	P10			
E. coli CFT073 *imp4213*	GBM	4×	8	6	5	9	12	8	15	11	8	5	6.1 × 10^10^	1.4 × 10^−9^	0.002
		8×	0	1	0	0	0	0	0	0	0	0	6.1 × 10^10^	3.8 × 10^−11^	LOS
E. coli CFT073	G0790	4×	31	30	19	22	20	18	11	16	26	25	7.5 × 10^10^	2.9 × 10^−9^	0.002
		8×	0	0	1	0	0	2	0	0	3	0	7.5 × 10^10^	1.6 × 10^−11^	LOS
E. coli CFT073 Δ*lpp*	G0790	4×	1	0	0	0	1	0	0	0	0	0	5.8 × 10^10^	3.4 × 10^−11^	LOS
		8×	0	0	0	0	0	0	0	0	0	0	5.8 × 10^10^	<1.7 × 10^−11^	LOS
E. cloacae 13047	G0790	4×	5	3	3	5	3	4	5	6	3	4	1.5 × 10^10^	2.7 × 10^−9^	0.002
		8×	1	0	0	0	0	0	0	0	0	0	1.5 × 10^10^	1.5 × 10^−10^	LOS
K. pneumoniae 700603	G0790	4×	279	202	205	191	192	212	225	209	212	217	4.5 × 10^10^	4.8 × 10^−8^	0.002
		8×	ND^*c*^										ND	ND	ND

a FOR, frequency of resistance. For strains which had no resistant colonies growing on plates, the *p*_0_ method was used to determine the FOR as discussed in Materials and Methods.

b *P* values were calculated using a one-sample Wilcoxon rank test comparing to the limit of sensitivity (LOS) of the FOR assay.

c ND, not done.

To determine if the complete loss of Lpp expression is the major *lpp*-dependent resistance mechanism to G0790 in CFT073, we picked 10 or 6 independent G0790-resistant CFT073 mutant strains from MHII agarose plates containing either 4× MIC or 8× MICs of G0790, respectively (Fig. 2B and C), and 2 independent G0790-resistant CFT073 Δ*lpp* strains (Fig. 2D) and performed Illumina short-read whole-genome sequencing (WGS) and Western blot analysis using an anti-Lpp rabbit polyclonal antibody (43). No Lpp expression was detected in several resistant strains selected from 4× MIC (4×-R4 and 4×-R9) and 8× MIC (8×-R3, 8×-R4, and 8×-R6) G0790-containing plates (Fig. 2B and C). WGS analyses of these mutants showed that 4×-R4, 4×-R9, 8×-R3, 8×-R4, and 8×-R6 all contained either complete or partial deletions of *lpp*. Genomic deletions encompassing *lpp* ranged from 6.2 kb (8×-R3) to 16.8 kb (8×-R4) (Fig. 2E; see also Fig. S2). While a subset of cells from 4×-R5, 4×-R8, and 8×-R2 contained complete *lpp* deletions, the patterns of relative Illumina read coverage across the regions surrounding *lpp* suggested they were made up of mixed populations with WT CFT073 upon initial isolation and have not been followed up in this study. The remaining G0790-resistant CFT073 mutant strains expressed Lpp at either lower or normal levels compared to that in WT CFT073 (Fig. 2B and C), indicating that complete deletion of *lpp* was not the only mechanism of G0790 resistance.

10.1128/mBio.02018-20.2 FIG S2 E. coli CFT073 gene map surrounding *lpp* showing depth of mapped Illumina reads for G0790-resistant mutants. The shaded green area indicates the position of *lpp*. Underlined strains contain *lspA*_GA. Download FIG S2, PDF file, 1.0 MB. Copyright © 2020 Pantua et al. 2020 Pantua et al. This content is distributed under the terms of the Creative Commons Attribution 4.0 International license.

**FIG 3:**
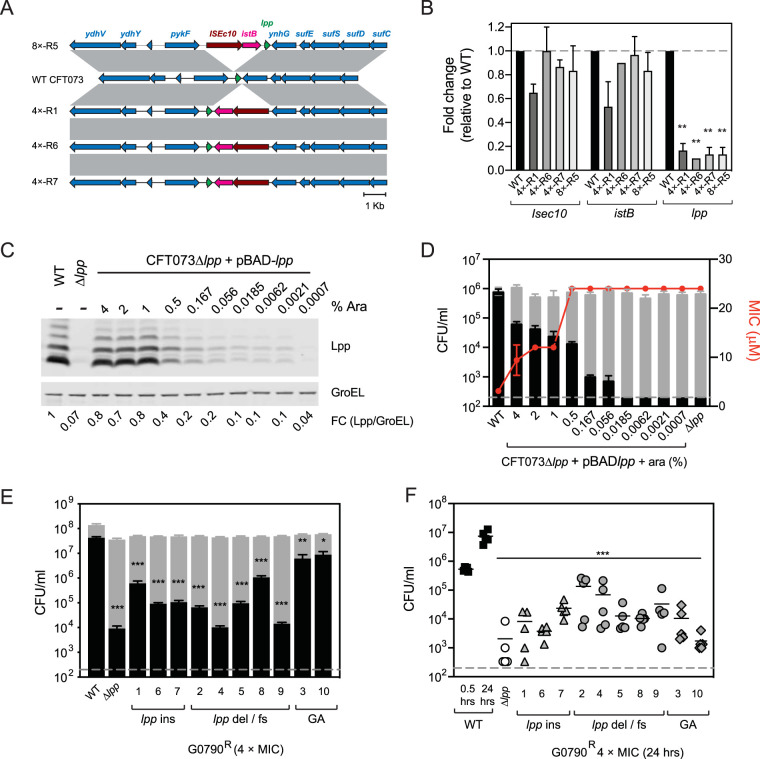
Insertion of IS*Ec*10-*istB* upstream of downstream of *lpp* confers G0790 resistance by downregulation of *lpp* gene expression. (A) Gene maps showing the IS*Ec*10-*istB* insertions upstream (8×-R5) and downstream (4×-R1, 4×-R6, and 4×-R7) of *lpp* in G0790-resistant strains. Pairwise BLAST identities are indicated by gray shading and show conserved regions among the strains. (B) Gene expression of IS*Ec*10, *istB*, and *lpp* in G0790-resistant strains containing *lpp* insertions detected by RT-qPCR. Relative quantitation of IS*Ec*10, *istB*, and *lpp* gene expression was calculated using the 2^−ΔΔ^*^CT^* method and normalized to *rrsB*. Results are graphed relative to WT CFT073 (set at 1, dotted line). These data are representative of two independent experiments, each performed in triplicates. **, *P* = 0.0016 for 4×-R1; **, *P* = 0.0015 for 4×-R6, 4×-R7, and 8×-R5. (C and D) Correlation of Lpp expression to serum killing and G0790 resistance. Lpp expression in CFT073 Δ*lpp* expressing an arabinose-inducible copy of *lpp* [CFT073 Δ*lpp*(pBAD-*lpp*)]. CFT073 Δ*lpp*(pBAD-*lpp*) was cultured with a range of arabinose concentrations, and cells were tested for expression of Lpp (C) and sensitivity to serum killing (D). Fold changes (FC) of Lpp expression normalized to GroEL are denoted (with Lpp/GroEL levels in WT CFT073 set at 1). In panel D, cells were treated with normal (black bars) or heat-inactivated (gray bars) human serum for 60 min, and enumerated CFU are plotted. ***, *P* < 0.001 for all normal human serum treatments compared to WT CFT073). *y* axis with MIC values graphed as a red line corresponds to the MIC values for each arabinose treatment. Dashed gray line represents the sensitivity of this assay (200 CFU/ml). (E) Sensitivity to normal (black bars) or heat-inactivated (gray bars) human serum of the 10 G0790-resistant strains identified from the 4× MIC G0790 resistance selections. *, *P* = 0.002; **, *P* = 0.0234; ***, *P* < 0.001. G0790-resistant strains have been grouped according to the type of mutations: *lpp* insertions (ins), *lpp* deletions or frameshift (del/fs) and genome amplification (GA). Dashed gray line represents the sensitivity of this assay (200 CFU/ml). (F) Intravenous infection of neutropenic C57BL/6 mice with WT CFT073 (black), CFT073Δ*lpp* (open circles), or G0790-resistant strains selected at 4× MIC (gray filled symbols). At 2 h and 24 h postinfection, bacterial burdens in the liver were enumerated. G0790-resistant strains have been grouped according to the type of mutations (*lpp* ins [gray triangles], *lpp* del/fs [gray circles], and GA [gray diamonds]). Pairwise comparisons were performed on log-normalized CFU and analyzed using one-way ANOVA with Dunnett’s multiple-comparison *P* value adjustment. ***, *P* < 0.001. The gray dashed line represents the limit of detection for this experiment (200 CFU/ml).

Multiple G0790-resistant strains expressed lower levels of Lpp than WT CFT073 (Fig. 2B and C). 4×-R2 contain a frameshift mutation at Asp54, which led to a significant reduction in expression of WT Lpp (Fig. 2F). Other CFT073 G0790-resistant strains such as 4×-R6 and 4×-R7 expressed ∼10- to 20-fold lower Lpp levels than WT CFT073 (Fig. 2B and C) and contained no additional SNPs or small indels in their genomes, suggesting that downregulation of Lpp may lead to G0790 resistance. WGS results identified insertions of IS*Ec10*-*istB* downstream (CFT073 4×-R1, 4×-R6, and 4×-R7) or upstream (CFT073 8×-R5) of the *lpp* coding region (Fig. 3A and Fig. S2). Reverse transcription-quantitative PCR (RT-qPCR) analyses demonstrated that insertion of the IS*Ec10*-*istB* locus either upstream or downstream of *lpp* leads to a ∼6- to 10-fold decrease in *lpp* gene expression (Fig. 3B). Using the proposed ∼500,000 Lpp molecules expressed per E. coli cell (44), this would mean that cells expressing ∼25,000 to 50,000 Lpp molecules would still be resistant to G0790. To determine the minimal levels of Lpp that still confer sensitivity to G0790, we used a previously generated CFT073 Δ*lpp* strain containing a plasmid that expresses an arabinose-inducible *lpp.* This allowed us to titrate levels of Lpp and measure G0790 activity and sensitivity to human serum killing, which is also dependent on Lpp (43). While high arabinose concentrations (1% to 4%) rescued Lpp expression levels in CFT073 Δ*lpp* and conferred resistance to serum killing similar to what was detected with WT CFT073, expression of ∼20% to 40% of WT CFT073 Lpp levels was sufficient to confer resistance to G0790 and led to increased sensitivity to serum killing (Fig. 3C and D). All G0790-resistant mutants containing *lpp* modifications were more sensitive to serum killing and attenuated *in vivo* (Fig. 3E and F). From these data, we conclude an ∼70% reduction of Lpp levels in E. coli CFT073 is sufficient to confer serum sensitivity and *in vivo* attenuation to levels seen in cells with a complete *lpp* deletion.

### Unstable genomic amplifications lead to G0790 resistance.

**FIG 4:**
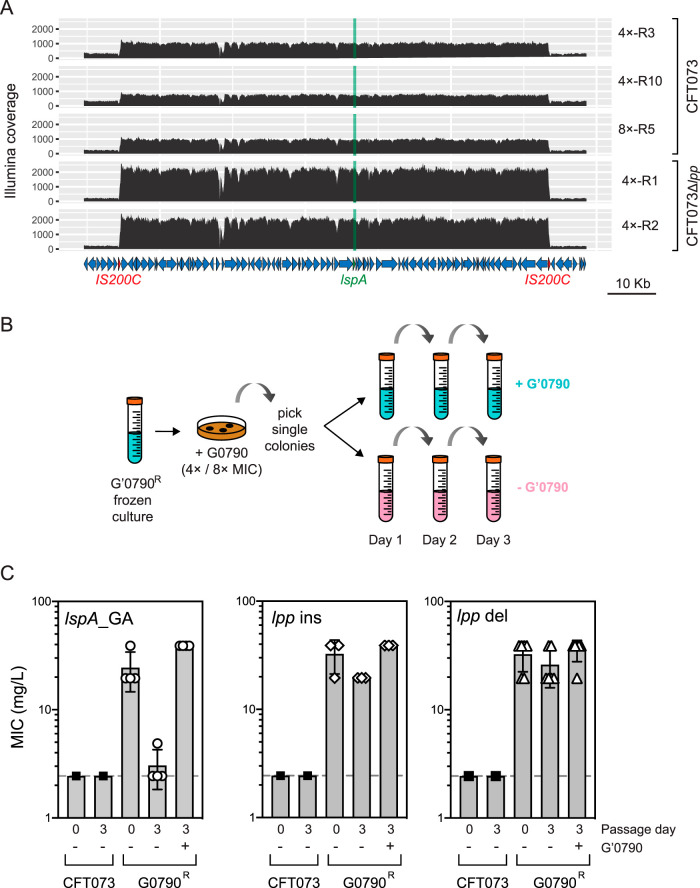
Passaging of G0790-resistant strains containing GA in the absence of G0790 leads to loss of G0790 resistance. (A) Illumina coverage of *lspA* and surrounding genomic region in 4×-R3, 4×-R10, 8×-R5, Δ*lpp* 4×-R1, and Δ*lpp* 4×-R2. Regions with higher relative coverage than the surrounding region correspond to amplified DNA. The *lspA* gene is shown in green, and repeat sequences flanking the amplified region are indicated in red. (B) Schematic describing the passaging of G0790-resistant strains. G0790-resistant strains from either 4× or 8× MIC MHII agarose plates were passaged over 3 days in the presence (blue) or absence (pink) of G0790, details for which are included in Materials and Methods. (C) G0790 resistance in strains containing *lspA*_GA is lost after passaging for 3 days in the absence of G0790. Shown here are graphed MIC values (in mg/liter) after 3 days of passaging in the presence and absence of G0790 of WT CFT073 (■) strains containing *lspA*_GA (○; 4×-R3, 4×-R10, 8×-R1, and 8×-R5), *lpp* insertions (◊; 4×-R1, 4×-R6 and 4×-R7), or lpp deletions (△; 4×-R2, 4×-R4, 4×-R9, 4×-R1, 8×-R3, 8×-R4, and 8×-R6). Each symbol corresponds to an individual G0790-resistant strain, and the dotted lines correspond to the MIC of WT CFT073 (2.44 mg/liter). MIC data are averaged from duplicate wells and taken from two independent experiments.

Four of the G0790-resistant mutants (4×-R3, 4×-R10, 8×-R1, and 8×-R5) showed no evidence of *lpp* modification and expressed normal levels of Lpp compared to that in WT CFT073 (Fig. 2B and C and Table 3; Fig. S2). In addition, even though CFT073 Δ*lpp* was more resistant to G0790 than the parental WT strain (Table 1), we were able to select two CFT073 Δ*lpp* mutants that were more resistant to G0790 than the parental CFT073 Δ*lpp* strain and did not identify any additional SNPs or indels in these mutants (Table 3). The decreased serum sensitivity of 4×-R3 and 4×-R10 relative to mutants containing *lpp* modifications (Fig. 3E), together with decreased susceptibility levels observed in the CFT073 Δ*lpp* mutants (4×-R1 and 4×-R2) (Table 3 and Fig. 2C), suggests resistance can be mediated by an Lpp-independent mechanism. WGS analyses identified multiple genomic amplifications (GAs) in G0790-resistant strains expressing normal levels of Lpp (Table 3; see also Fig. S3). The GAs ranged in size from 89 kb to 3 Mb, and in most cases, they are flanked by large direct sequence repeats, suggesting homologous recombination likely was involved in their initial formation (45–47) (Fig. 4A; see also Table S1 and Fig. S2). Here, based on whether or not the GAs contained the *lspA* gene, we refer to them as *lspA*_GA or non-*lspA*_GA, respectively (Fig. S3). While both *lspA*_GA and non-*lspA*_GA can be found in some mutants, those strains that only contained non-*lspA*_GA (CFT073 4×-R3/R4/R5 and 8×-R3/R6) also contained *lpp*-related modifications that led to either a partial or complete loss of Lpp expression (Table 3 and Fig. S2). In contrast, no significant differences were detected between the Lpp levels in the mutants containing *lspA*_GA and in WT CFT073 (Fig. 2B and C) (Mann-Whitney test *P* = 0.095). These data suggest that the *lspA*_GA may be important for conferring G0790 resistance in cells that do not have *lpp* modifications.

**TABLE 3:** Identified genomic alterations in G0790-resistant E. coli CFT073 and CFT073 Δ*lpp* strains by whole-genome sequencing

Strain background	Strain name	*Lpp* modification	*lspA* copy no.^*a*^	Genomic amplification^*b*^	MIC (mg/liter)
CFT073	Parent		1.1		2.44
	4×-R1	IS*Ec10*-*istB* insertion (3′ of *lpp*)	1.2		19.5
	4×-R2	Lpp p.Asp54fs^*c*^	1.3	Non-*lspA_*GA	19.5
	4×-R3		6.7	*lspA_*GA	19.5
	4×-R4	*lpp* deletion	1.1	Non-*lspA_*GA	19.5
	4×-R6	IS*Ec10*-*istB* insertion (3′ of *lpp*)	1.0		39
	4×-R7	IS*Ec10*-*istB* insertion (3′ of *lpp*)	1.2	Non-*lspA_*GA	39
	4×-R9	*lpp* deletion	1.2		19.5
	4×-R10		5.2	*lspA_*GA	19.5
				
	8×-R1		4.1	*lspA_*GA	19.5
	8×-R3	5′ *lpp* deletion	1.1	Non-*lspA_*GA	39
	8×-R4	*lpp* deletion	1.1		39
	8×-R5	IS*Ec10*-*istB* insertion (5′ of *lpp*)	4.4	*lspA_*GA	39
	8×-R6	*lpp* deletion	1.4	Non-*lspA_*GA	19.5
CFT073 Δ*lpp*	Parent	Constructed *lpp* deletion	1.0		19.5
	4×-R1	Constructed *lpp* deletion	11.0	*lspA_*GA; non-*lspA_*GA	78
	4×-R2	Constructed *lpp* deletion	10.0	*lspA_*GA; non-*lspA_*GA	78

a Copy number estimated using Illumina sequencing coverage.

b *lspA_*GA refers to genomics amplifications that encompass *lspA*, and non-*lspA_*GA refers to amplifications that do not encompass *lspA.*

c fs, frameshift mutation.

10.1128/mBio.02018-20.3 FIG S3 WGS analyses of G0790-resistant strains identify two major regions of genomic amplification. Illumina coverage plots showing paired reads aligned across the entire CFT073 genome. G0790-resistant strains were compared to their respective parental strains. Two distinct regions of amplification denoted as *lspA*_GA and non-*lspA*_GA in the manuscript are color coded yellow and cyan, respectively. The genomic locations of *lspA* and *lpp* are denoted by the red and blue lines, respectively. For ease of presentation, the genomic positions have been offset 2.5 Mb so that amplifications are not split. Download FIG S3, PDF file, 1.7 MB. Copyright © 2020 Pantua et al. 2020 Pantua et al. This content is distributed under the terms of the Creative Commons Attribution 4.0 International license.

10.1128/mBio.02018-20.7 TABLE S1 Genomic amplifications in G0790 mutants that were subsequently subjected to passage as inferred using whole-genome sequencing. Download Table S1, DOCX file, 0.1 MB. Copyright © 2020 Pantua et al. 2020 Pantua et al. This content is distributed under the terms of the Creative Commons Attribution 4.0 International license.

As GAs are known to be highly unstable (45, 48), we wanted to test the stability of *lspA*_GA. The G0790-resistant mutants containing GAs were selected on MHII agarose plates containing G0790 and passaged for 3 days in the presence or absence of G0790 (Fig. 4B). As controls, we also passaged G0790-resistant mutants containing *lpp* insertions or *lpp* deletions. Lpp and LspA protein expression and G0790 sensitivity were measured daily, and WGS analysis was performed on bacterial populations recovered after 3 days. Lpp and LspA total protein levels did not change significantly in cell lysates after passaging in the presence or absence of G0790 (see Fig. S5). Resistant strains containing IS*Ec10*-*istB* insertions downstream of *lpp*, or in which *lpp* was deleted, maintained resistance to G0790 after passaging in the absence of G0790 (Fig. 4C; see also Fig. S4A and B). In contrast, MIC values for the G0790-resistant CFT073 mutants containing the *lspA*_GAs (4×-R3, 4×-R10, 8×-R1, and 8×-R5) passaged in the absence of G0790 were comparable to the MIC of the parental WT strain (Fig. 4C). The 8×-R5 strain, which after initial selection contained a GA and an IS*Ec10*-*istB* insertion upstream of *lpp*, reverted to the WT phenotype when passaged in the absence of G0790 (Fig. 4). WGS confirmed that while *lspA*_GAs were maintained after passaging in the presence of G0790, they were lost when passaging in the absence of G0790 (see Fig. S6). Overall, these data show that both *lspA*_GA and non-*lspA*_GA are highly unstable in the absence of G0790 and that their loss confers sensitivity to G0790.

10.1128/mBio.02018-20.4 FIG S4 Loss of G0790-resistant phenotype after passaging for 4 days in the absence of G0790. G0790-resistant strains containing *lpp* insertions (A), *lpp* deletions of frameshifts (B), or GAs (C) were passaged in the presence (blue) or absence (red) of G0790. MIC values of the overnight culture were measured after each day of passaging. Particular G0790-resistant strain names are listed. For comparison, MIC values of CFT073 (black) passaged in the absence of G0790 are graphed. For G0790-resistant strains containing *lpp* deletions, MIC values of the CFT073 Δ*lpp* (gray) were graphed. Download FIG S4, PDF file, 0.2 MB. Copyright © 2020 Pantua et al. 2020 Pantua et al. This content is distributed under the terms of the Creative Commons Attribution 4.0 International license.

10.1128/mBio.02018-20.5 FIG S5 Lpp and LspA protein expression after passaging for 4 days in the presence or absence of G0790. Total cell lysates were harvested from G0790-resistant CFT073 (A and B) or CFT073 Δ*lpp* (C) cells after 3 days of passaging in the presence or absence of G0790, and Western blot analyses were performed to detect levels of Lpp and LspA. Blots were probed for GroEL as a loading control. Fold changes (FC) of LspA and Lpp expression were quantitated by normalizing to GroEL levels and compared to each respective parental strain (set at 1). Data are representative of two independent experiments. Download FIG S5, PDF file, 0.9 MB. Copyright © 2020 Pantua et al. 2020 Pantua et al. This content is distributed under the terms of the Creative Commons Attribution 4.0 International license.

10.1128/mBio.02018-20.6 FIG S6 Passaging G0790-resistant strains containing *lspA*_GA in the absence of G0790 results in loss of GA. G0790-resistant strains from CFT073 (4×-R3, 4×-R10, 8×-R1, and 8×-R5), CFT073 Δ*lpp* (4×-R1 and 4×-R2), and their parental strains were passaged in the presence (+) or absence (−) of G0790 for 3 days, and the Illumina coverage plots showing paired reads were aligned across the entire CFT073 genome. The two distinct regions of amplification denoted as *lspA*_GA and non-*lspA*_GA in the manuscript are color coded yellow and cyan, respectively. The genomic locations of *lspA* and *lpp* are denoted by the red and blue lines, respectively. Genomic positions have been offset 2.5 Mb so that amplifications are not split. Download FIG S6, PDF file, 1.6 MB. Copyright © 2020 Pantua et al. 2020 Pantua et al. This content is distributed under the terms of the Creative Commons Attribution 4.0 International license.

### *lspA*_GA confers G0790 heteroresistance via moderate upregulation of LspA protein levels.

**FIG 5:**
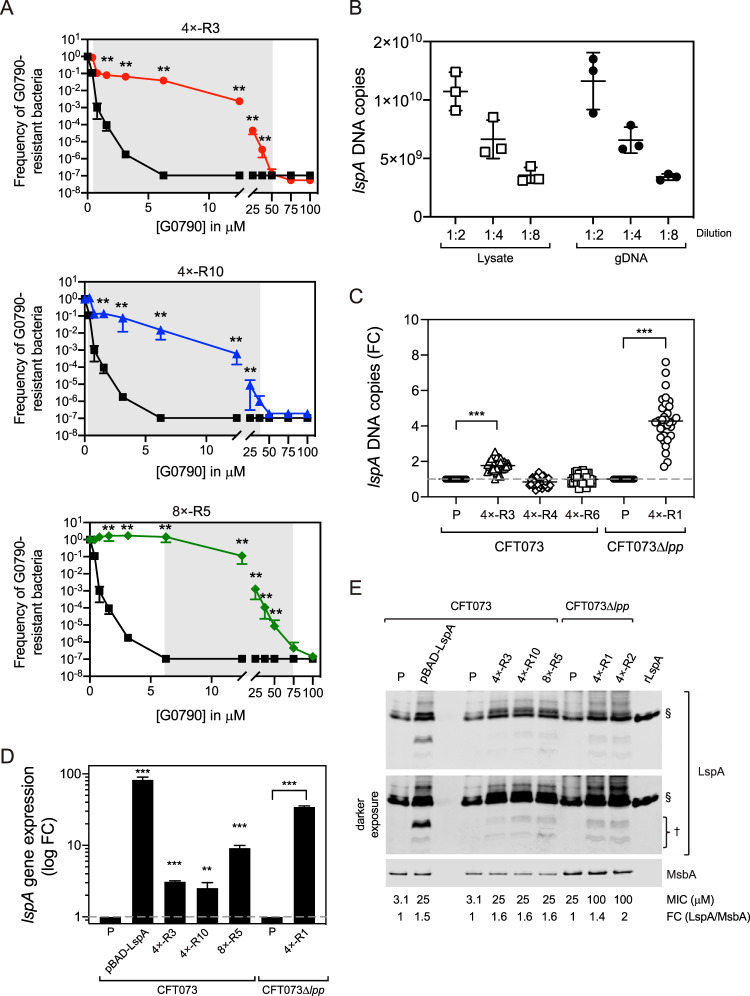
Unstable heteroresistance caused by genomic amplifications is mediated by modest upregulation of LspA protein levels in the inner membrane. (A) Population analysis profiles of G0790-resistant strains containing GAs confirm the heteroresistance phenotype. Population analysis profile analyses were performed on WT CFT073 (black) and CFT073 4×-R3 (red), 4×-R10 (blue), and 8×-R5 (green) by enumerating CFU growing on MHII agarose plates containing a range of G0790 concentrations. Heteroresistance is defined if there is a > 8-fold difference between the highest noninhibitory concentration and the highest inhibitory concentration, which is shaded gray in the graphs. Ratios of CFU/total CFU per plate were calculated and graphed on the *y* axis (total CFU per plate for WT CFT073, CFT073 4×-R3, 4×-R10, and 8×-R5 were 1.9 × 10^9^, 3.7 × 10^9^, 1.03 × 10^9^, and 4.7 × 10^9^ CFU, respectively). The sensitivity of the assay was 200 CFU/ml. These data (means ± standard deviations [SDs]) are representative of two independent experiments each performed in triplicates. **, *P* = 0.0022 by Mann-Whitney test. (B) Comparison of quantitative PCR analysis of *lspA* DNA using cell lysates versus genomic DNA. Three independent CFT073 colonies were picked and grown to mid-exponential phase (OD_600_ of 0.7), and each culture was split to generate bacterial lysates or purified genomic DNA (gDNA) (as detailed in Materials and Methods). qPCR was performed using primers specific to *lspA*. *lspA* copy numbers were calculated using a standard curve generated using a plasmid containing lspA. These data (means ± SDs) are representative of two independent experiments each performed in triplicates. (C) Quantitative PCR analysis of *lspA* DNA copy number in G0790-resistant representative strains for CFT073 (GA, 4×-R3; *lpp* insertion, 4×-R6; *lpp* deletion, 4×-R4) and CFT073 Δ*lpp* (GA, 4×-R1). Single colonies for each strain were picked, and relative quantitation of *lspA* expression using the 2^−ΔΔ^*^CT^* method was performed. Fold changes (FC) were calculated by normalizing *lspA C_T_* values to those for *lepB*, which is not encompassed in the genomic amplifications, and compared to their respective parental strains (P), which are set at 1. ***, *P* < 0.0001. (D) *lspA* gene expression in G0790-heteroresistant strains was measured by RT-qPCR in WT CFT073, CFT073 Δ*lpp*, or G0790-resistant strains containing GA. Relative quantitation of *lspA* expression using the 2^−ΔΔ^*^CT^* method is graphed as fold changes (FC) calculated by normalizing *lspA C_T_* values to those for *lepB*, which is not encompassed in the genomic amplifications, and compared to their respective parental strains, which was set at 1. ***, *P* < 0.0001; **, *P* = 0.0049. These data are representative of two independent experiments each performed in triplicates. (E) LspA protein levels in the inner membrane of G0790-heteroresistant and parent strains. Inner membranes were isolated from WT CFT073, CFT073 Δ*lpp*, or G0790-resistant strains containing *lspA*_GA, and LspA protein levels were measured by Western blotting. Fold changes (FC) of LspA expression were quantitated by normalizing to MsbA levels and by comparing to each respective WT parental strain (set at 1). MIC values for each strain are denoted below the Western blot images. Recombinant LspA (rLspA) was loaded as a control to confirm the correct band for quantitation. These data are representative of at least two independent experiments.

Given the instability of the GAs, we wanted to formally test for heteroresistance using the population analysis profile method, which is the gold standard assay for identifying heteroresistance (2). Cells from each culture were plated on different drug concentrations, and the fraction of cells that survived was determined by enumerating CFU. A mutant was considered heteroresistant if the antibiotic concentration exhibiting the highest inhibitory effects was at least 8-fold higher than the highest noninhibitory concentration (2). We performed the population analysis profile assays on 4×-R3, 4×-R10, and 8×-R5, which are the G0790-resistant strains containing *lspA*_GA. We generated three independent cultures each for CFT073, 4×-R3, 4×-R10, and 8×-R5, plated them on different concentrations of G0790, and enumerated the CFU. The fold differences between the highest inhibitory concentrations and highest noninhibitory concentrations were 128-fold, 96-fold, and 12-fold for 4×-R3, 4×-R10, and 8×-R5, respectively (shaded gray areas in Fig. 5A), consistent with the hypothesis of heteroresistance.

The heteroresistance phenotype likely results from different levels of *lspA* amplification among subpopulations of cells in culture. To test this, we picked 32 individual colonies each from G0790-resistant strains containing *lspA*_GA (CFT073 4×-R3 and CFT073 Δ*lpp* 4×-R1), *lpp* deletion (4×-R4), or *lpp* insertion (4×-R6) and quantitated *lspA* DNA copies directly from cell lysates. First, we confirmed that quantitation of *lspA* copy numbers from lysates was comparable to levels detected using purified genomic DNA (gDNA) (Fig. 5B). qPCR analysis of colonies isolated from the heteroresistant populations contained a higher distribution of *lspA* DNA levels (ranges of 1 to 2.55 and 1.7 to 7.6 *lspA* copies for CFT073 4×-R3 and CFT073 Δ*lpp* 4×-R1, respectively) than the parental strains (Fig. 5C). In contrast, levels of *lspA* DNA in the resistant populations containing *lpp* deletions (4×-R4) or *lpp* insertions (4×-R6) were comparable to those seen in WT CFT073 (Fig. 5C). Furthermore, CFT073 Δ*lpp* 4×-R1, which contained the highest *lspA* Illumina read coverage, also contained the highest level of *lspA* DNA levels (Fig. 5C). These data suggest that a higher *lspA* copy number mediates G0790 resistance by increasing LspA protein levels.

Plasmid-based *lspA* overexpression has been demonstrated to confer resistance to GBM and TA (30, 49), suggesting that the GA encompassing *lspA* could similarly lead to resistance through a gene dosage effect. To test this hypothesis, we compared *lspA* DNA copy number to *lspA* mRNA and protein levels in G0790 heteroresistant strains by RT-qPCR and Western blot analyses, respectively. We decided to measure LspA protein levels in the bacterial inner membrane where it is localized and active, because we did not detect any notable changes in total cellular LspA protein levels in the initially isolated resistant strains (Fig. 2) or the passaging experiments (Fig. S5). We used CFT073 cells containing a plasmid overexpressing LspA [CFT073(pBAD*lspA*)], which is known to lead to G0790 resistance, as a control (Table 1). While *lspA* mRNA levels correlated with the number of *lspA*_GAs, LspA protein levels in the inner membrane were only increased by a modest ∼1.5- to 2-fold in all heteroresistant strains tested compared to that in their parental strains, irrespective of the *lspA* transcript levels (Fig. 5D and E). While CFT073 4×-R3 and CFT073 Δ*lpp* 4×-R1 differed in their *lspA* transcriptional levels by ∼11-fold, they both overexpressed LspA to similar extents (1.4- to 1.6-fold) (Fig. 5D and E). CFT073(pBAD*lspA*), which showed an ∼83-fold increase in *lspA* gene expression compared to that in CFT073 cells without the plasmid, also overexpressed LspA by only ∼1.5-fold, similar to that seen with the G0790 heteroresistant strains (Fig. 5D and E), confirming a modest overexpression of LspA is sufficient to confer G0790 resistance. Western blot analyses revealed the presence of lower-molecular-weight forms of LspA in cells overexpressing LspA which were absent in the respective parental strains (Fig. 5E). These results demonstrate that the G0790 heteroresistance phenotype in cells containing *lspA*_GA is mediated by moderate overexpression of LspA protein levels in the inner membrane.

## DISCUSSION

Our efforts to identify potent GBM analogs were driven by whole bacterial cell activity assays due to the low translatability of *in vitro* biochemical activity (34, 35). This strategy led to the early identification of G0790, which has ∼13-fold increased WT E. coli activity but similar *in vitro* biochemical potency compared to that of GBM. The GBM 50% inhibitory concentration (IC_50_) previously described by Dev et al. (GBM *K_i_* = 36 nM) (20) is much higher than what we have described and may be the result of lower specific activity of their LspA enzyme preparation due to the lack of detergent matrix in their biochemical assays. Given G0790 is still more active than GBM against the outer membrane-permeable strain CFT073 *imp4213*, the increase in G0790 whole-cell activity is likely due to a combination of increased penetration through the outer membrane and higher target acquisition. Published data have demonstrated that resistance can occur via either deletion of *lpp* or removal of the C-terminal lysine that eliminates the peptidoglycan-linked Lpp form (31). This is consistent with CFT073 4×-R2, which contained a frameshift mutation in Lpp (p.Asp54fs) (Fig. 2), which would lead to a mutant protein that does not contain the C-terminal lysine required for linkage to peptidoglycan. Interestingly, expression of WT Lpp is significantly decreased in CFT073 4×-R2, suggesting the mutant Lpp protein is either highly unstable or not recognized by the anti-Lpp antibody we are using. Furthermore, our data show that even a modest decrease in Lpp protein levels leads to levels of resistance comparable to that of *lpp*-deleted strains (Table 3 and Fig. 3). Decreased Lpp levels were caused by insertion of IS*Ec10*-*istB* either up- or downstream of the *lpp* gene. Insertion of IS*Ec10*, a putative insertion sequence (IS) element, into the promoter or attenuator regions of the chromosomal *ampC* gene has been demonstrated to cause AmpC overexpression and confer resistance to third-generation cephalosporins (50). *istB* encodes a transposon nucleoside triphosphate (NTP)-binding protein which catalyzes transposition of IS elements (51). This was unexpected given that these G0790-resistant cells containing the *lpp* insertions are estimated to still express ∼120,000 to 180,000 Lpp molecules per cell. One possible explanation for these results is that the decreased level of Lpp does not lead to a significant accumulation of the Lpp-peptidoglycan toxic intermediate in the inner membrane. Alternatively, there may be a preferential loss of the peptidoglycan-associated form of Lpp in the G0790-resistant strains containing the *lpp* insertions. The passaging experiments indicate that resistance in strains containing IS*Ec10*-*istB* insertions is stable in the absence of G0790 (Fig. 4B). In either scenario, our data suggest that E. coli CFT073 efficiently regulates the number of Lpp molecules needed for optimal cellular growth and virulence.

Mechanisms of subpopulation antibiotic resistance such as persistence, tolerance, and heteroresistance are increasingly being associated with antibiotic failure against clinical isolates (52). Transient heteroresistance can sometimes be lost after a single culture in the absence of selection (53), which suggests that they may go largely undiagnosed based on current practices in clinical laboratories. The *lspA* amplifications presented in this study are highly unstable and were lost after as few as two subcultures in the absence of G0790. Although the mutants were highly resistant to begin with, this resistance was identified using tightly controlled *in vitro* FOR assays with no subculturing in the absence of G0790 after selection from the plates. In contrast, in a clinical setting where one or more subcultures of an isolate in the absence of selection is a likely scenario, it is conceivable that the amplification could be rapidly lost, leading to the misclassification of the strain as antibiotic susceptible and subsequently increasing the risk of inappropriate treatment. While heteroresistance frequencies are estimated to be ∼10^−5^ to 10^−6^ (54), our FOR data for GBM and G0790 are significantly lower (Table 2). Heteroresistance was first attributed to GA in 1977 in a β-lactamase-hyperproducing E. coli K-12 strain (55). Since then, multiple mechanisms of gene duplication have been implicated in heteroresistance phenotypes (56). The GA identified in G0790-heteroresistant strains are mostly flanked by tandem repeat sequences (Fig. 4C; see also Table S1 in the supplemental material), consistent with homologous recombination as the mechanism of formation. The unstable G0790 heteroresistance phenotype is unstable in the absence of selection, likely due to fitness costs associated with amplifying large portions of the bacterial genome (Fig. 3F). This is consistent with previous reports in multiple clinical Gram-negative bacterial isolates (11). Our data clearly indicate that *lspA*_GA is the mechanism underlying the observed G0790 heteroresistance, as LspA protein levels in the strains containing the *lspA*_GA are equivalent to those observed in cells that contain only an *lspA*-overexpressing plasmid (Fig. 5D). Therefore, a modest level of LspA overexpression in the inner membrane is sufficient to confer G0790 resistance. Some heteroresistant strains contained both *lspA*_GA and non-*lspA*_GA (CFT073 4×-R3/R10 and CFT073 Δ*lpp* 4×-R1/R2) (Table 3), but the role of the non-*lspA*_GA is unclear and awaits further investigation. While most G0790-resistant strains containing both *lspA*_GA and non-*lspA*_GA maintained both amplifications after passaging in the presence of G0790, others maintained only one or the other (CFT073 4×-R3/8×-R1) (Fig. S3 and S6). Interestingly, the strains that contained only the non-*lspA*_GA also contained insertions, gene deletions, or point mutations in or around the *lpp* gene. Our data suggest that the *lspA*_GA is sufficient to mediate resistance to G0790, as CFT073 8×-R5, which only contains the *lspA*_GA (Fig. S3B), shows a heteroresistance phenotype with a similar MIC shift to those of other G0790-resistant cells that contain both *lspA*_GA and non-*lspA*_GA. The non-*lspA*_GA in CFT073 Δ*lpp* 4×-R1 is much larger and inclusive of that detected in CFT073 Δ*lpp* 4×-R2 (Fig. S3C), hinting at a minimal genomic region for further investigation. While we cannot definitely rule out that the non-*lspA*_GAs, which are maintained in the presence of G0790, could be playing a role in resistance to LspA inhibitors, our cumulative data lead us to hypothesize that the non-*lspA*_GA may be important in compensating for defects in outer membrane integrity or bacterial fitness due to the deletion or reduced expression of Lpp, especially when maturation of other lipoproteins is also inhibited by G0790.

While our data suggest that the mechanism by which *lspA*_GAs confer G0790 heteroresistance is by overexpressing LspA protein levels, we detected only a modest 1.5- to 2-fold increase in LspA protein levels in the inner membrane. We conclude that this results in G0790 heteroresistance, as we detected equivalent increases in LspA levels in parental CFT073 cells that only contained an *lspA*-overexpressing plasmid, suggesting this modest overexpression is sufficient to lead to G0790 resistance. Furthermore, LspA protein levels did not correlate with DNA or mRNA copy number (Fig. 5), and levels of *lspA* gene expression far exceeded that of LspA protein expression. While the lack of correlation between mRNA and protein abundances in prokaryotic and eukaryotic systems is well known (57), this discordance between mRNA and protein levels after LspA overexpression remains an intriguing yet unexplained finding. One explanation could be that there is a fitness cost associated with higher LspA overexpression, which is consistent with published data demonstrating that overexpression of bacterial inner membrane proteins often leads to bacterial cell toxicity (58, 59). What is interesting about our data is that the bacterial cells seem to tolerate much less *lspA* overexpression in the inner membrane than overexpression of other inner membrane proteins, suggesting that E. coli CFT073 may have mechanisms to efficiently regulate LspA levels in the inner membrane. We also detected lower-molecular-weight LspA forms by Western blot analyses in G0790-resistant cells containing *lspA*_GAs or containing an *lspA* plasmid but not in the parental strains (Fig. 5D). While the mechanism leading to the generation of these lower-molecular-weight LspA forms is currently unknown and needs further examination, one explanation could be that significant LspA overexpression may lead to the activation of stress response pathways that induce proteases to regulate levels of full-length LspA in the inner membrane (60). Whether higher levels of overexpression are tolerated in other clinical E. coli isolates is unclear and would warrant further study. Our data seem to suggest that E. coli CFT073 tightly regulates *lspA* overexpression but allows for sufficient overexpression to confer resistance to inhibitors of LspA.

Our data build on previous findings of unstable heteroresistance in clinical isolates to provide further rationale for profiling mechanisms of heteroresistance during preclinical evaluation of antibiotic candidates. Moreover, our comprehensive characterization of diverse mechanisms of resistance to LspA inhibitors emphasizes the importance of GA in generating heteroresistance. Our data further demonstrate that all G0790-resistant mutants identified in this study have decreased pathogenicity *in vivo*, which bodes well from the perspective of resistance evolution to LspA inhibitors in the clinics. Given that GAs are unstable and can potentially be lost prior to antibiotic susceptibility testing during routine subculturing, it will be crucial for clinical laboratories to adapt their operating procedures to readily detect heteroresistance in patient clinical isolates.

## MATERIALS AND METHODS

### Ethics statement.

All mice used in this study were housed and maintained at Genentech in accordance with American Association of Laboratory Animal Care guidelines. All experimental studies were conducted under protocol 17-2630 approved by the Institutional Animal Care and Use Committee of Genentech Laboratory Animal Research, an Association for Assessment and Accreditation of Laboratory Animal Care (AAALAC) International-accredited facility in accordance with the Guide for the Care and Use of Laboratory Animals and applicable laws and regulations.

### Bacterial strains, media, and compounds.

The bacterial strains and isolates used in this work are described in Table S2 in the supplemental material. Bacteria were cultured in cation-adjusted Mueller-Hinton II (MHBII) or Luria-Bertani medium. MG1655, an E. coli K-12 strain, and CFT073, a clinical uropathogenic E. coli clinical strain, were used for the majority of the experiments. MG1655 containing a conditional deletion of *lspA* (MG1655 Δ*lspA*) was constructed according to a previously described method (43, 61). Briefly, the arabinose-inducible *lspA* from pBAD24 was subcloned into the SacI site of the integration vector pLDR9. The NotI-digested and religated construct was integrated into the lambda *att* site using the lambda integrase. The endogenous *lspA* gene was then replaced with a kanamycin marker flanked by FLP recombination target (FRT) sites generated using primers MG1655 Δ*lspA-*F and MG1655 Δ*lspA-*R (see Table S3) and integrated into MG1655 by lambda Red recombinase-mediated homologous recombination (62). Unless stated otherwise, all antibiotics were obtained from Sigma-Aldrich. G0790 was synthesized by the Genentech Chemistry Department. Stocks for all compounds or antibiotics used in this work were prepared fresh at 10 mM concentrations in dimethyl sulfoxide (DMSO) and diluted for use in experiments.

10.1128/mBio.02018-20.8 TABLE S2 Bacterial strains and plasmids used in this study. Download Table S2, DOC file, 0.1 MB. Copyright © 2020 Pantua et al. 2020 Pantua et al. This content is distributed under the terms of the Creative Commons Attribution 4.0 International license.

10.1128/mBio.02018-20.9 TABLE S3 Primers used in this study for strain generation and quantitative PCR. Download Table S3, DOC file, 0.1 MB. Copyright © 2020 Pantua et al. 2020 Pantua et al. This content is distributed under the terms of the Creative Commons Attribution 4.0 International license.

### Antibodies.

Antibodies against E. coli Lpp, MsbA, and BamA have been previously described (43, 63–65). The anti-LspA antibody was generated using an LspA protein and purified similarly to the anti-Lpp antibody. The GroEL antibody was obtained from ENZO Life Sciences. Secondary antibodies with IRdye for immunoblot detection were purchased from LI-COR.

### MIC, *in vitro* growth, and serum killing assays.

MICs of compounds in each strain were determined in cation-adjusted Mueller-Hinton II (MHII) broth or MHII agar according to the Clinical and Laboratory Standards Institute protocol (CLSI 2006). To measure the effect of protein binding on activity, MIC assays were performed in the presence of 50% heat-inactivated human serum. The serum killing assay was performed as described previously (43). FOR assays and growth on plates containing G0790 were always performed on MHII agarose plates.

To determine if *lspA* from A. baumannii 17978 or 19606 can rescue growth of the MG1655 Δ*lspA* inducible deletion strain, MG1655 Δ*lspA* cells were transformed with an empty vector (pLMG18) or pLMG18 expressing *lspA* from either E. coli MG1655, A. baumannii ATCC 17978, or A. baumannii ATCC 19606. Bacteria were grown overnight in LB broth containing 1% glucose and 12.5 μg/ml tetracycline and back diluted to ∼1 × 10^5^ CFU/ml in 5 ml LB broth containing 0.2% glucose such that all *lspA* expression occurred from pLMG18-encoded *lspA.* Cultures were incubated at 37°C, and CFU were enumerated at various times posttreatment.

### Molecular modeling of LspA in complex with G0790.

The crystal structure of LspA in complex with globomycin (PDB ID 5DIR) was used to build a model of G0790 bound to LspA. The coordinates were prepared in MOE (Chemical Computing Group) using the quick prep algorithm to protonate appropriate atoms, tether atoms in the vicinity of the ligand, and fix atoms further than 8 Å from the ligand. The complex was minimized before building a model of G0790 by building (*S*)-2,3-diaminopropionic acid, cyclohexylglycine, and *N*-methyl-norvaline at positions a, b, and c, respectively, before minimizing the complex again.

### Expression and purification of E. coli LspA and development of the LspA biochemical assay.

An E. coli LspA construct containing a noncleavable C-terminal 6×His tag was recombinantly expressed by autoinduction in BL21(DE3) cells at 16°C for 64 h. Cells were harvested by centrifugation at 4,500 × *g* and resuspended in lysis buffer [25 mM Tris (pH 7.5), 150 mM NaCl, 10% glycerol, 1 mM Tris(2-carboxyethyl)phosphine hydrochloride (TCEP), and 1× Roche EDTA-free protease inhibitor cocktail]. Cells were lysed by three passes through a microfluidizer at 10,000 lb/in^2^. Unlysed cells and debris were removed by centrifugation at 24,000 × *g* for 12 min, and the supernatant was further centrifuged at 125,000 × *g* for 1 h to isolate the membrane fraction. Membranes were resuspended in buffer A (25 mM Tris [pH 7.5], 150 mM NaCl, 20 mM imidazole, 10% glycerol, 1 mM TCEP, 1% lauryl maltose neopentyl glycol [LMNG]), the solution was stirred at 4°C for 1 h, and insoluble material was removed by centrifugation at 125,000 *g* for 1 h. The supernatant was incubated in a batch with nickel affinity resin overnight at 4°C and applied to a gravity column to collect the resin, and the resin was washed with 10 column volumes (CV) buffer A followed by 10 CV buffer B (25 mM Tris [pH 7.5], 150 mM NaCl, 40 mM imidazole, 10% glycerol, 1 mM TCEP, 0.05% LMNG). Bound protein was eluted with 3 CV buffer B containing 300 mM imidazole. Eluate was concentrated to 1.5 ml and applied to a Superdex 200 16/60 column that had been preequilibrated in buffer C (25 mM Tris [pH 7.5], 150 mM NaCl, 10% glycerol, 1 mM TCEP, 0.05% LMNG). Fractions containing pure LspA were pooled and concentrated to 1 mg/ml.

The LspA enzymatic activity was measured by liquid chromatography-mass spectrometry (LC-MS) detection of the peptide product. The peptide substrate (diacylglycerol [DAG]-Pal-biotin; Anaspec) is based on the E. coli lipoprotein Pal with the following sequence: MQLNKV-L(U^13^C_6_,^15^N)-KGL(U13C6,15N)MIALPVMAIAA-dipalmitoyl_2_C-SSNKNGG-K-biotin, which upon cleavage by LspA, yields the product peptide dipalmitoyl_2_C-SSNKNGG-K-biotin. A product standard [dipalmitoyl_2_C-SSNKNAAK-(NHCH2CH2NH)-biotin; CPC Scientific] was in the reaction mixture as an internal standard for normalization of product quantitation. The standard assay consists of a 25-μl reaction mixture with 0.25 nM LspA-LMNG (Anatrace), 10 μM DAG-Pal-biotin, 0.5 μM product standard in 50 mM Tris (pH 7.5), 100 mM NaCl, 1 mM TCEP, 0.02% LMNG, and 0.01% bovine skin gelatin. The reaction is quenched after 3 h at 37°C with 25 μl of formic acid. The quenched mixture was analyzed by LC-MS (Waters ultraperformance liquid chromatography [UPLC] charged-surface hybrid [CSH] C_18_, 0% to100% acetonitrile in 0.1% formic acid; Sciex QTRAP 6500), and the product peak area under the concentration-time curve (AUC) was normalized with that of the internal standard.

### Visualization of G0790-treated CFT073 by time-lapse and transmission electron microscopy.

Electron microscopy was performed as previously described (61). For time-lapse microscopy, CFT073 cells expressing cytoplasmic green fluorescent protein (GFP) were grown to exponential phase in MHB supplemented with 10 μg/ml gentamicin and treated with 12.4 μM G0790 (corresponding to 4× MIC). Cells were immediately placed between a coverslip and a 1% MHII agarose pad containing 12.4 μM G0790 and 10 μg/ml gentamicin for imaging. Cells were maintained at 37°C during imaging with a stage-top chamber (Okolab Inc.). Cells were imaged on a Nikon Eclipse Ti inverted confocal microscope (Nikon Instruments Inc.) coupled with an UltraVIEW VoX (PerkinElmer Inc.) and a 100× (numerical aperture [NA] 1.40) oil-immersion lens objective. Images were captured at various times using an ORCA-Flash 4.0 CMOS camera (Hamamatsu Photonics), collected using Volocity software (Quorum Technologies), and processed using Fiji (66).

### Purification of peptidoglycan-associated proteins.

Purification of peptidoglycan-associated proteins (PAPs) was performed according to published methods (43, 67, 68) with some modifications. Briefly, bacteria were harvested in mid-exponential phase for treatment and then subjected to PAP extraction by resuspending cell pellets from an optical density (OD; *A*_600_) of 10 in 6 ml of PAP extraction buffer containing 2% (wt/vol) SDS in 100 mM Tris-HCl (pH 8.0) with 100 mM NaCl, 10% glycerol, and cOmplete mini EDTA-free protease inhibitor cocktail (Sigma-Aldrich). After 60 min at room temperature (RT), the extraction was subjected to centrifugation at 100,000 × *g* for 60 min at 22°C, and the pellet, containing peptidoglycan-associated proteins, was washed once with the same PAP extraction buffer with centrifugation at 100,000 × *g* for 30 min and resuspended in 200 μl of PAP extraction buffer (referred to as the SDS-insoluble or PAP fraction). The supernatant containing the SDS-soluble fraction was aliquoted and frozen (referred to as the non-PAP fraction). Both fractions were treated with equal volumes of BugBuster buffer prior to the addition of sample buffer for Western immunoblotting as described above.

### Isolation of E. coli inner and outer membranes.

The inner and outer membrane fractionations of bacterial cells were performed according to published methods (69), with some modifications. Bacterial pellets (approximately 10 OD/ml) were resuspended in 2 ml resuspension buffer containing 25 mM HEPES and protease inhibitor (Roche). The bacterial suspensions were then homogenized by passing them through a microfluidizer (Microfluidics LV1) twice. The homogenates were then cleared by centrifugation at 4,500 × *g* at 4°C for 10 min. To isolate the total membranes, the cleared homogenates were transferred into ultracentrifuge tubes (Beckman 355647) and spun at 230,000 × *g* using Beckman Optima Max XP ultracentrifuge and TLA 100.3 rotor at 4°C for 1 h. The pellet contains the total membrane, while the supernatant contains the periplasmic and cytoplasmic fractions. The supernatants were collected, and the pellets were gently washed with 0.2 ml resuspension buffer. The total membrane pellets were then resuspended in 0.5 ml fractionation buffer containing 2% sodium lauryl sarcosinate (Sarkosyl, IBI Scientific IB07080), 25 mM HEPES, and protease inhibitor, incubated at room temperature on a nutator for 30 min, and transferred to ultracentrifugation tubes (Beckman 343778). The inner membrane (IM) and OM were separated by ultracentrifugation (Rotor TLA 120.2) at 230,000 × *g* at room temperature for 1 h. The supernatants which contained the solubilized inner membrane were collected by pipetting approximately 0.4 ml. The pellets which contained the outer membrane fraction were washed gently and resuspended in 0.1 ml resuspension buffer. All samples were processed for SDS-PAGE by combining with lysis buffer and incubating at RT for 10 min prior to addition of sample buffer.

### SDS-PAGE and Western immunoblotting.

Bacterial cells were lysed in lysis buffer (30 μl of BugBuster, 3 μl Benzonase, 1 μl lysozyme and protease inhibitors), and proteins were separated by SDS-PAGE using 16% tricine or 10% to 20% Tris glycine resolving gels (Thermo Fisher Scientific). Proteins were transferred to nitrocellulose membranes using the iBlot 2 gel horizontal transfer system (Invitrogen) and blocked using LI-COR Odyssey phosphate-buffered saline (PBS) blocking buffer for 30 min. Primary antibodies were diluted as follows in PBS containing 0.05% Tween 20 and 1× blocking buffer and incubated with the membranes overnight at 4°C: rabbit anti-Lpp polyclonal antibody (1:10,000 final dilution), rabbit anti-LspA polyclonal antibody (1:2,000 final dilution), rabbit anti-GroEL polyclonal antibody (1:10,000), rat anti-BamA 29E9 monoclonal antibody (1:5,000 final dilution), and rabbit anti-MsbA polyclonal antibody (1:1,000). Nitrocellulose membranes were washed twice in PBS containing 0.05% Tween 20 for 15 min each and incubated for 1 h at RT with secondary antibodies obtained from LI-COR, used as per the manufacturer’s instructions. Images were collected using the Odyssey CLx imaging system (LI-COR) and analyzed by Image Studio Lite.

### Frequency of resistance assays and isolation of G0790-resistant strains.

Frequencies of resistance (FORs) of G0790 were determined for E. coli CFT073, E. cloacae (ATCC 13047), and K. pneumoniae (ATCC 700603) as previously published (70). To determine the FOR, 3 to 4 colonies of the strain to be tested were picked from a fresh plate and diluted to an optical density at 600 nm (OD_600_) of 0.00001 in 1 ml of cation-adjusted MHBII. Ten independent 5-ml cultures in MHBII were prepared by inoculating 5 μl (approximately 1 × 10^3^ CFU) and incubated at 37°C for 18 h. The bacteria were pelleted by centrifugation at 4000 × *g* at 4°C for 15 min and resuspended in 0.5 ml fresh MHBII. Two hundred fifty microliters of the bacterial suspension was plated evenly on MHII agarose plates containing globomycin or G0790 at a final concentration of 4× and 8× MIC. In parallel, CFU from each independent culture was measured by spotting 10-fold serial dilutions on MHII agar plates and incubated at 37°C for 18 h. Colonies were counted at days 1, 2, and 3 postinoculation. FOR was calculated as the ratio of the total number of resistant colonies relative to the total CFU plated. For strains that had no resistant colonies growing on plates, the *p*_0_ method was used to calculate the FOR (FOR = m/total CFU plated, where m = −ln *p*_0_ and *p*_0_ is the proportion of cultures with no mutants; 0.7 ≥ *p*_0_ ≥ 0.1), as described previously (71). Colonies were picked and resuspended in 30 μl MHBII broth medium, and 10 μl was plated on an MHII agar plate containing G0790 to be used to determine the MIC. Of the remaining 20 μl, half was used to inoculate 1.5 ml MHBII broth medium containing G0790 and grown at 37°C for 18 h to generate cell lysates for Western blot analysis, isolate genomic DNA (gDNA) for whole-genome sequencing, and for frozen glycerol stocks.

### Whole-genome sequencing of G0790-resistant strains.

Bacterial pellets grown from individual colonies were processed using the DNeasy Blood and Tissue kit (Qiagen) based on the manufacturer’s protocol. Whole-genome sequencing libraries were generated starting with 100 ng DNA input and using Nextera Flex DNA kit (Illumina) according to the manufacturer’s instructions. The size of the libraries was determined by 4200 TapeStation and high-sensitivity D1K screen tape (Agilent Technologies). The libraries were multiplexed and sequenced on an HiSeq 2500 (Illumina) to generate >200 million paired-end 75-bp reads per library. Reads were aligned to the E. coli CFT073 genome, and point mutations were detected as previously described (32). The sequences reported in this paper have been deposited in the NCBI Sequence Read Archive (accession nos. SAMN15889826–SAMN15889859 [BioProject PRJNA658823]).

### Detection of point mutations and structural variations.

Illumina paired-end reads were mapped onto the E. coli CFT073 reference genome (GenBank accession number CP051263) using GSNAP version 2013-10-10 (72). Single nucleotide variant detection was performed as previously described (32). For detection of large insertions and deletions, we implemented a two-phase strategy. First, we used an in-house R script that visualize discordant read pairs indicative of novel adjacencies. Assembly was then used to completely resolve sequence regions showing evidence of structural variation. Assembly was performed using the SPAdes version 3.10.1 (73) with default parameter setting. Bandage version 0.8.1 was used to visualize assembly graphs and identify connections among contigs. Gene maps and associated BLAST (74) comparisons were visualized using EasyFig (75).

Amplified regions were initially identified on the basis of abnormal read depth (1.5× mean sequencing depth) using custom R scripts. Regions with higher relative coverage than the surrounding region corresponded to amplified DNA. Amplification boundaries were verified using manual inspection of Integrative Genomics Viewer (IGV) (76) read pileups. The average copy number of *lspA* was estimated by dividing the sequence coverage of the *lspA* gene by the mean coverage for housekeeping genes *lepB* and *recA*. These genes were chosen for normalization in favor of the seven single-copy E. coli multilocus sequence type (MLST) genes (*adk*, *fumC*, *gyrB*, *icd*, *mdh*, *purA*, and *recA*) because they did not occur within amplified sequence regions in any of the mutants investigated.

### Mouse infection model.

Mouse infections to determine virulence of G0790-resistant and corresponding parent strains were evaluated in 7-week-old female neutropenic C57BL/6 mice (CR/Hollister) via an intravenous infection model. Bacterial inocula were prepared as described previously (43). Briefly, overnight bacterial cultures were back diluted 1:100 in M9 medium and grown at 37°C to mid-exponential phase (OD_600_ of 0.7). Cells were harvested, washed once with phosphate-buffered saline (PBS), and resuspended in PBS containing 10% glycerol. Aliquots of cells were frozen, and CFU from thawed aliquots were enumerated prior to mouse infections. The day of the mouse infection, aliquots were thawed and diluted to a final concentration of 1 × 10^6^ CFU per 100 μl PBS. Mice were rendered neutropenic by peritoneal injection of 2 doses of cyclophosphamide (150 mg/kg body weight on day −4 and 100 mg/kg on day −1). On day 0, mice were infected by intravenous injection through the tail vein with 1 × 10^6^ CFU mid-exponential-phase bacteria diluted in PBS. At 30 min and 24 h postinfection, bacterial burden in the liver and spleen was determined by serial dilutions of tissue homogenates on LB plates.

### *In vitro* passaging of G0790-resistant CFT073 and CFT073 Δ*lpp*.

Passaging of G0790-resistant and parent CFT073 and CFT073 Δ*lpp* strains was performed by inoculating 5 μl of the bacterial glycerol stock into 1.5 ml LB broth in the presence or absence of G0790 at 0.25× MIC for each respective strain and cultured at 37°C for 16 h. This bacterial culture is referred to as passage 1 (P1). Five-microliter aliquots from overnight P1 cultures were used to inoculate a fresh 1.5-ml culture of LB broth alone or LB broth containing 0.25× MIC G0790, incubated as mentioned above, and repeated to generate P2 and P3 cultures. For each passage, MICs were measured and cell lysates and genomic DNA were prepared for Western blot and whole-genome sequencing analyses, respectively.

### Titration of Lpp expression.

CFT073 Δ*lpp* cells expressing an arabinose inducible *lpp* (CFT073 Δ*lpp*::pBAD-*lpp*) were grown in different arabinose concentrations (4, 2, 1, 0.5, 0.1667, 0.0556, 0.0185, 0.0062, 0.0021, and 0.0007%) and carbenicillin (50 μg/ml) and incubated at 37°C for 8 h. For each of the 8-h cultures, MIC assays, Western blot analyses, and serum killing assays were performed. MIC assays were performed by adding 5 μl of the diluted 8-h cultures to the MIC assay.

### Reverse transcription and real-time PCR.

G0790-resistant strains and their respective parental strains were grown in triplicates to mid-exponential phase (OD_600_ of 0.5) in the presence of 0.25× MIC of G0790 for each strain. Total RNA was extracted from bacterial pellets by using an RNeasy kit (Qiagen) according to the manufacturer’s recommendations. cDNA synthesis was carried out using the high-capacity reverse transcription kit according to the manufacturer’s instruction (Applied Biosystem). Real-time PCR was performed using primers (2.5 μl of 1× IDT primer-probe reaction mix) (Table S2), cDNA (5 μl), and TaqMan Universal master mix in a 25-μl reaction mixture in the Applied Biosystem 7500 real-time PCR system. Relative expression was normalized to *rrsB* and calculated using the comparative threshold cycle (2^−ΔΔ^*^CT^*) method as described previously (77, 78).

To determine the levels of *lspA* DNA in the G0790-resistant strains, 1:10,000 dilutions of glycerol stocks of G0790-resistant and parental strains were plated on LB agar plates containing G0790 at either 4× or 8× MIC final concentrations and incubated at 37°C for 16 h. Approximately 32 individual colonies were picked and inoculated into LB broth containing G0790 and cultured to mid-exponential phase (OD_600_ of 0.7). Bacterial pellets were lysed in a lysis buffer containing 1× CutSmart buffer (New England Biolabs) and 2% Triton X-100 and incubated at 98°C for 40 s. Real-time PCR was performed using primers to *lspA* and *lepB* (Table S2) and TaqMan Universal master mix in a 25-μl reaction mixture as described above. Relative expression was calculated by normalizing to *lepB*, which is not located in the genomic amplified region, and calculated using the 2^−ΔΔ^*^CT^* method as described above.

### Population analysis profile.

The population analysis profiles of G0790 heteroresistant subpopulations were determined according to a method described previously (79). Briefly, three independent cultures for each G0790-resistant mutant strain were grown overnight at 37°C in MHBII containing G0790 at sub-MICs to maintain selective pressure. Five microliters or 10-fold serial dilution (10^−1^ to 10^−7^) in duplicates for each culture were transferred to MHII agarose plates or MHII agarose plates containing a range of G0790 concentrations (100, 75, 50, 37.5, 25, 12.5, 6.25, 3.125, 1.56, 0.8, and 0.4 μM). CFU were enumerated after incubation at 37°C for 18 h. Heteroresistance was defined using the criteria published by El-Halfawy and colleagues, which states there should be a >8-fold difference between the highest noninhibitory concentration and the highest inhibitory concentration (2, 80).

### Statistical analyses.

All statistical analyses were performed using GraphPad Prism software (GraphPad). Unless stated otherwise, all graphs represent the means ± the standard errors of the means (SEMs). Unless stated otherwise, *P* values for all data were determined using unpaired Mann-Whitney tests assuming the data were nonparametric. For the *in vivo* data, one-way analysis of variance (ANOVA) with Dunnett’s multiple-comparison *P* value adjustment was performed.
