# Supplementary figures and images for: Unstable Mechanisms of Resistance to Inhibitors of Escherichia coli Lipoprotein Signal Peptidase
Source: mBio. 2020 Sep 8;11(5):e02018-20. doi: 10.1128/mBio.02018-20 (PMC7482066; doi:10.1128/mBio.02018-20)

A

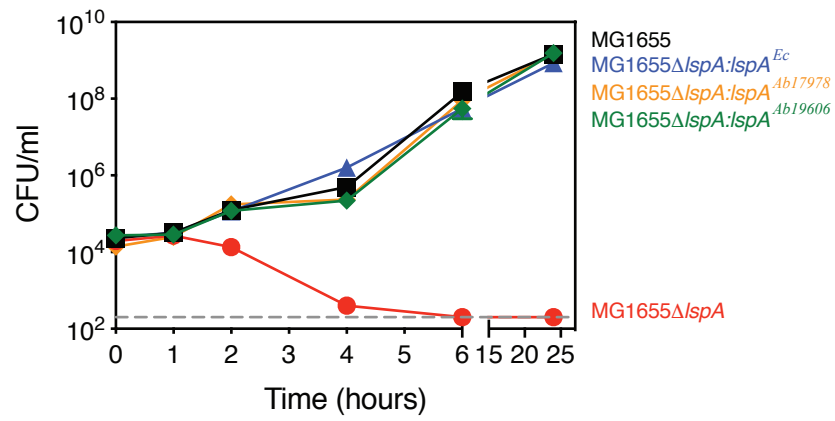

B

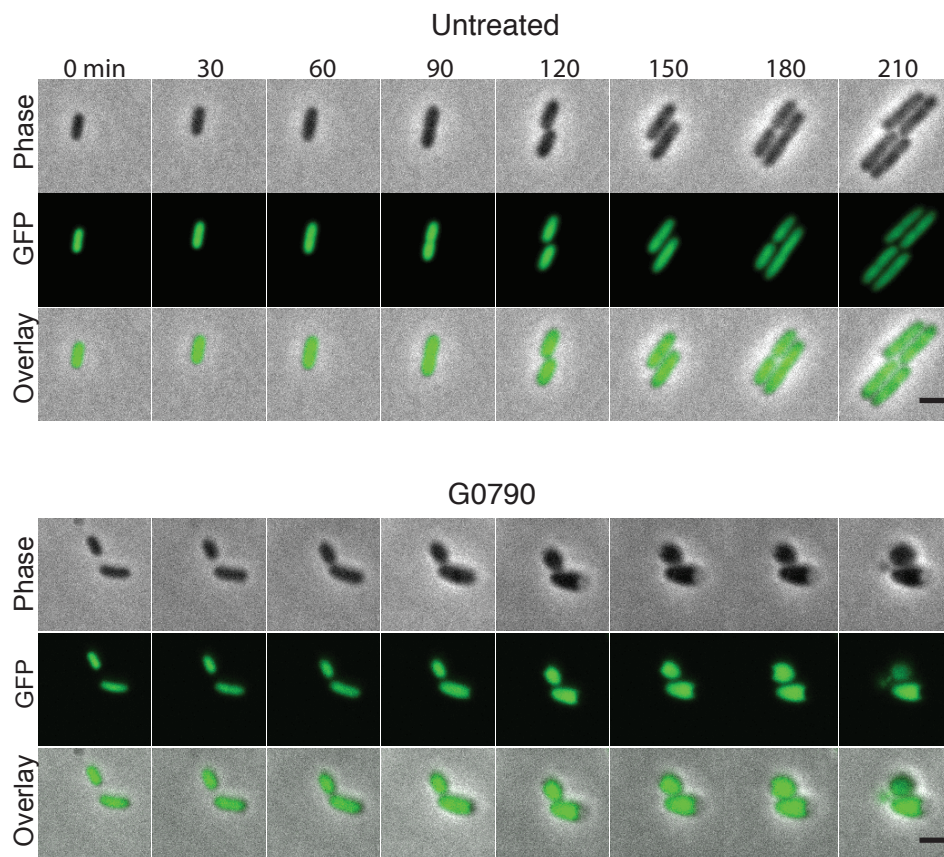

Figure S1

Supplement: FIG S1 [file mBio.02018-20-sf001.pdf]

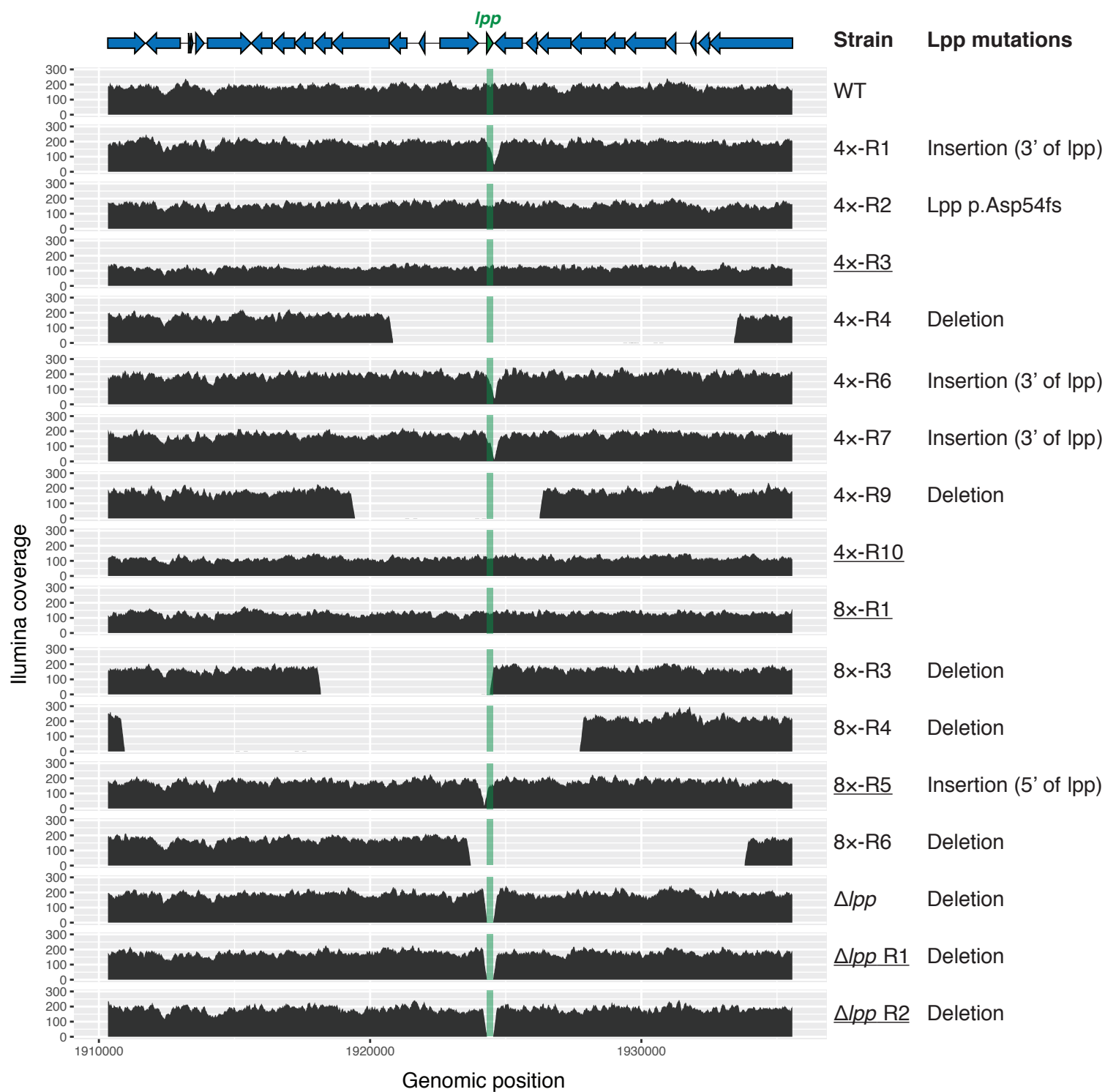

Underlined strains contain *lppA-GA*

Figure S2

Supplement: FIG S2 [file mBio.02018-20-sf002.pdf]

A

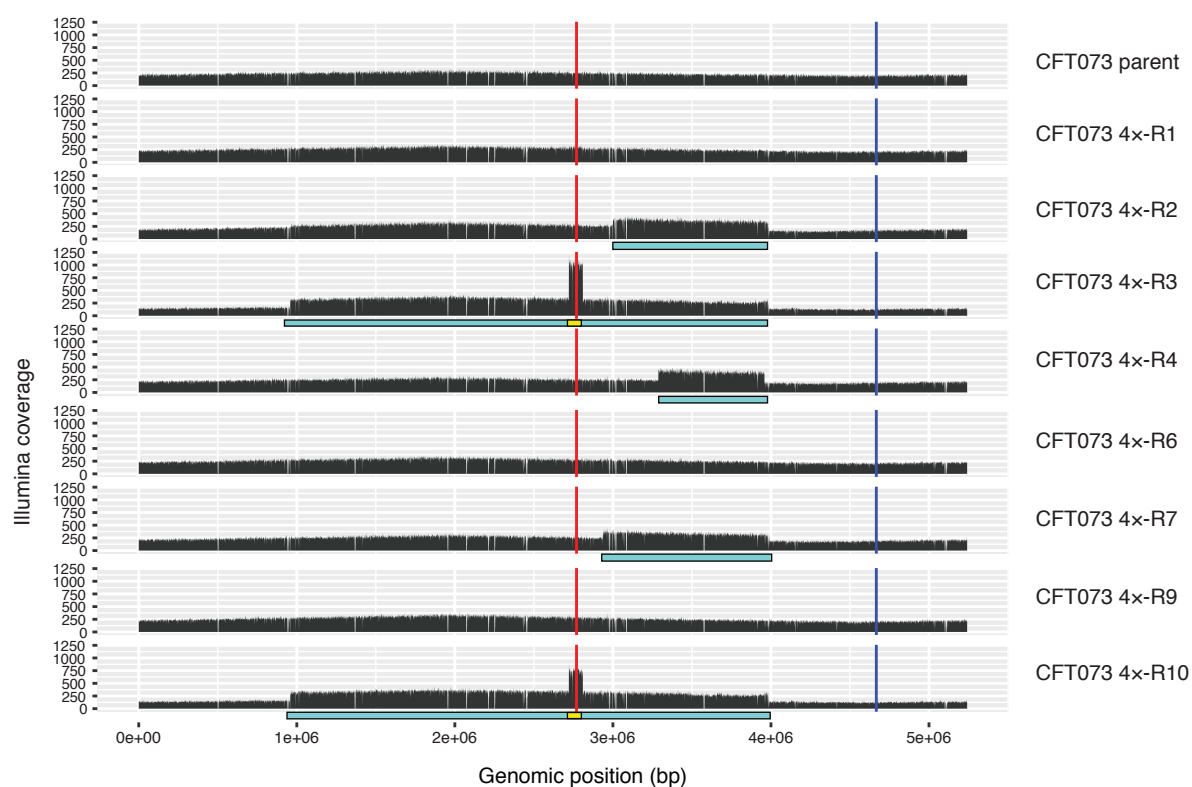

B

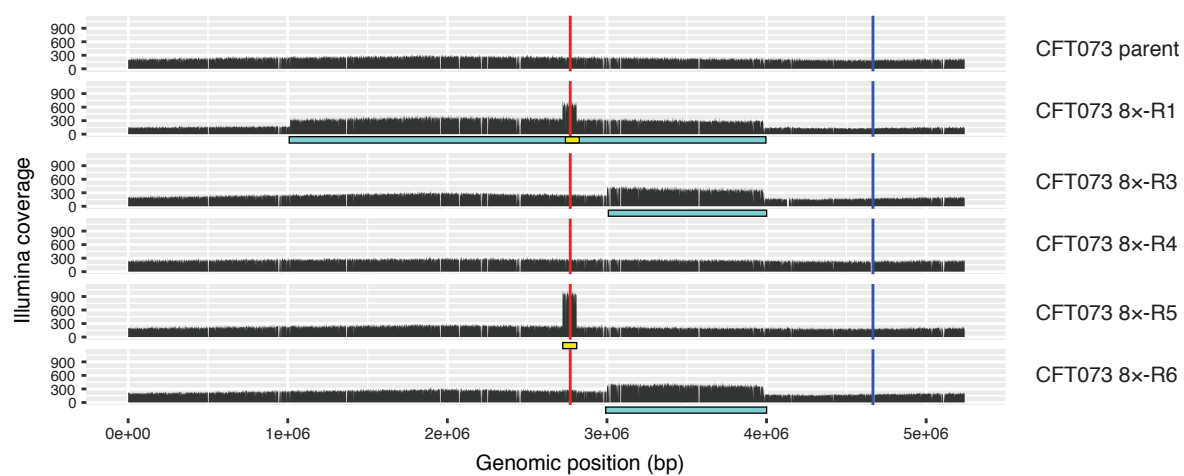

C

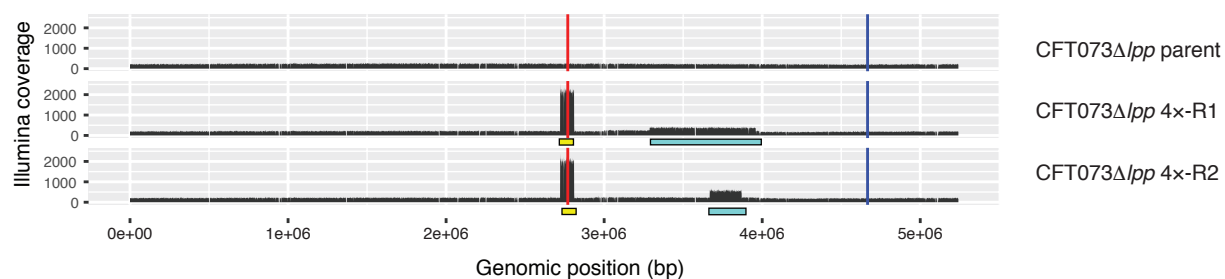

Figure S3

Supplement: FIG S3 [file mBio.02018-20-sf003.pdf]

A

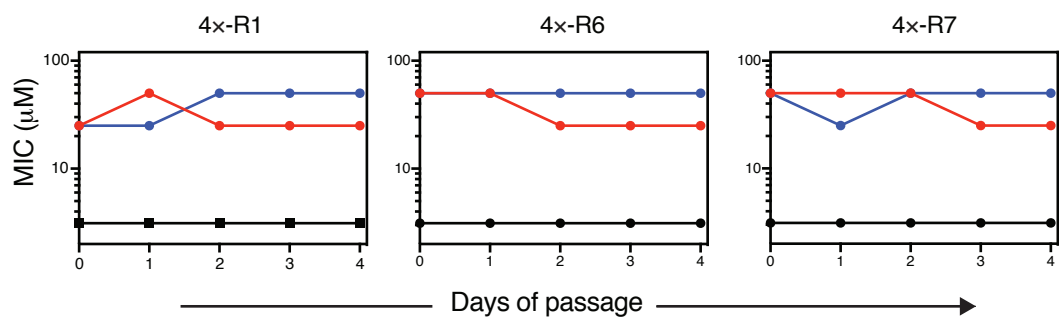

B

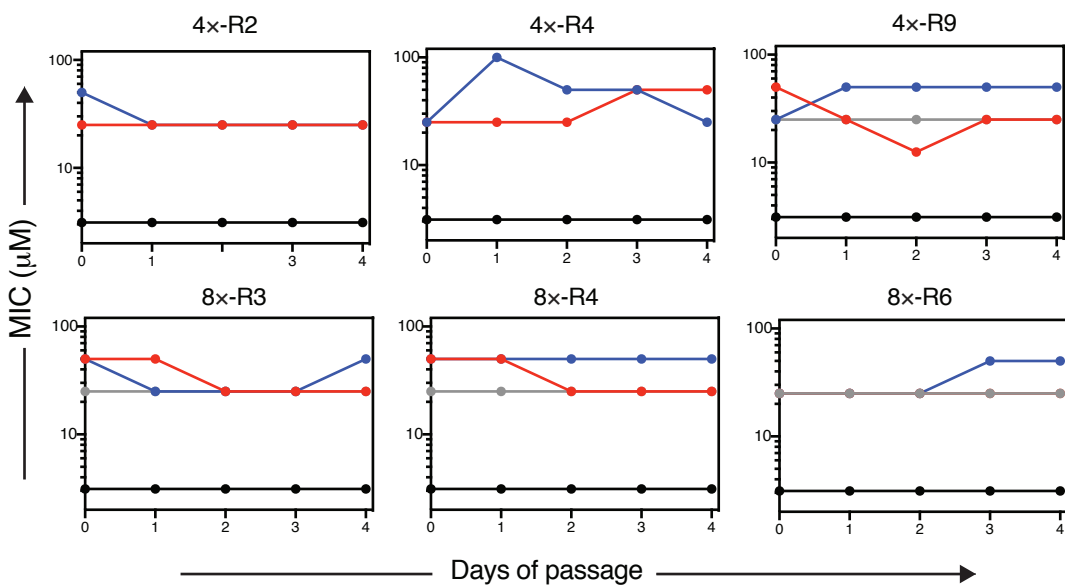

C

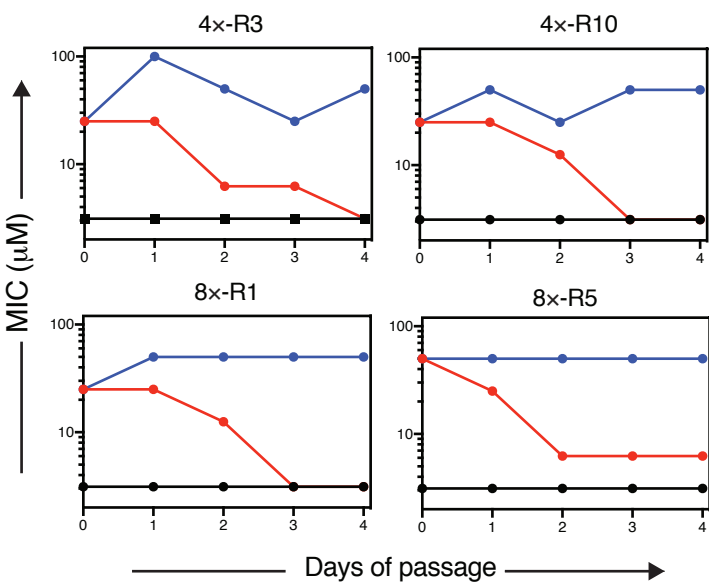

Figure S4

Supplement: FIG S4 [file mBio.02018-20-sf004.pdf]

A

**CFT073**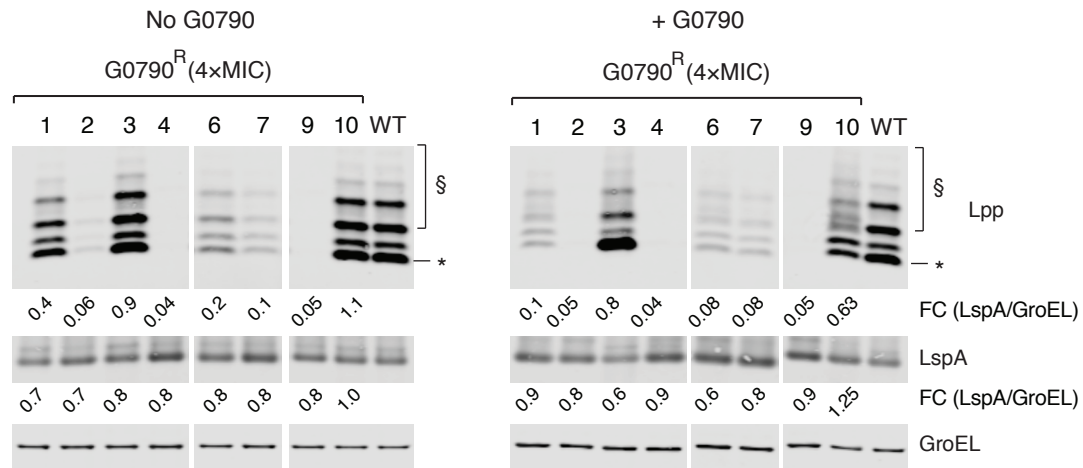

B

**CFT073**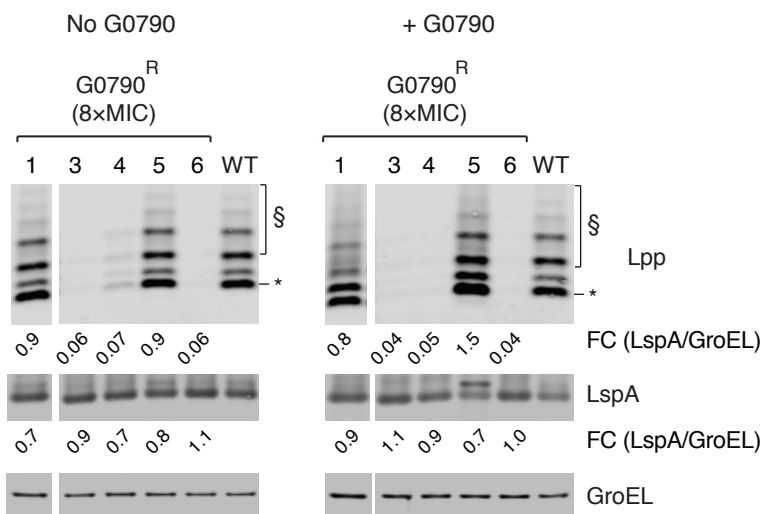

C

**CFT073Δlpp**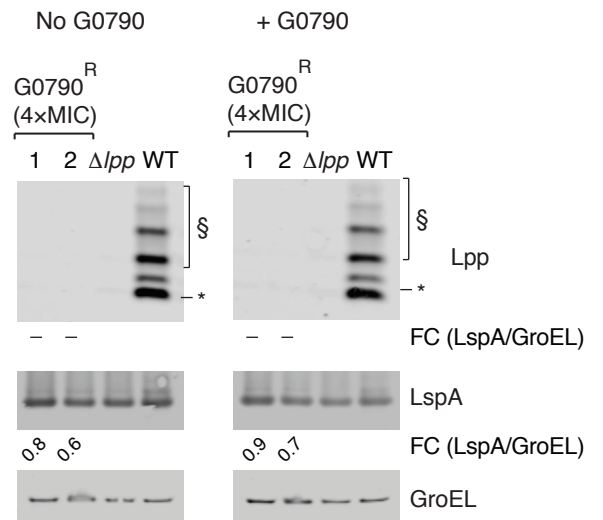

Figure S5

Supplement: FIG S5 [file mBio.02018-20-sf005.pdf]

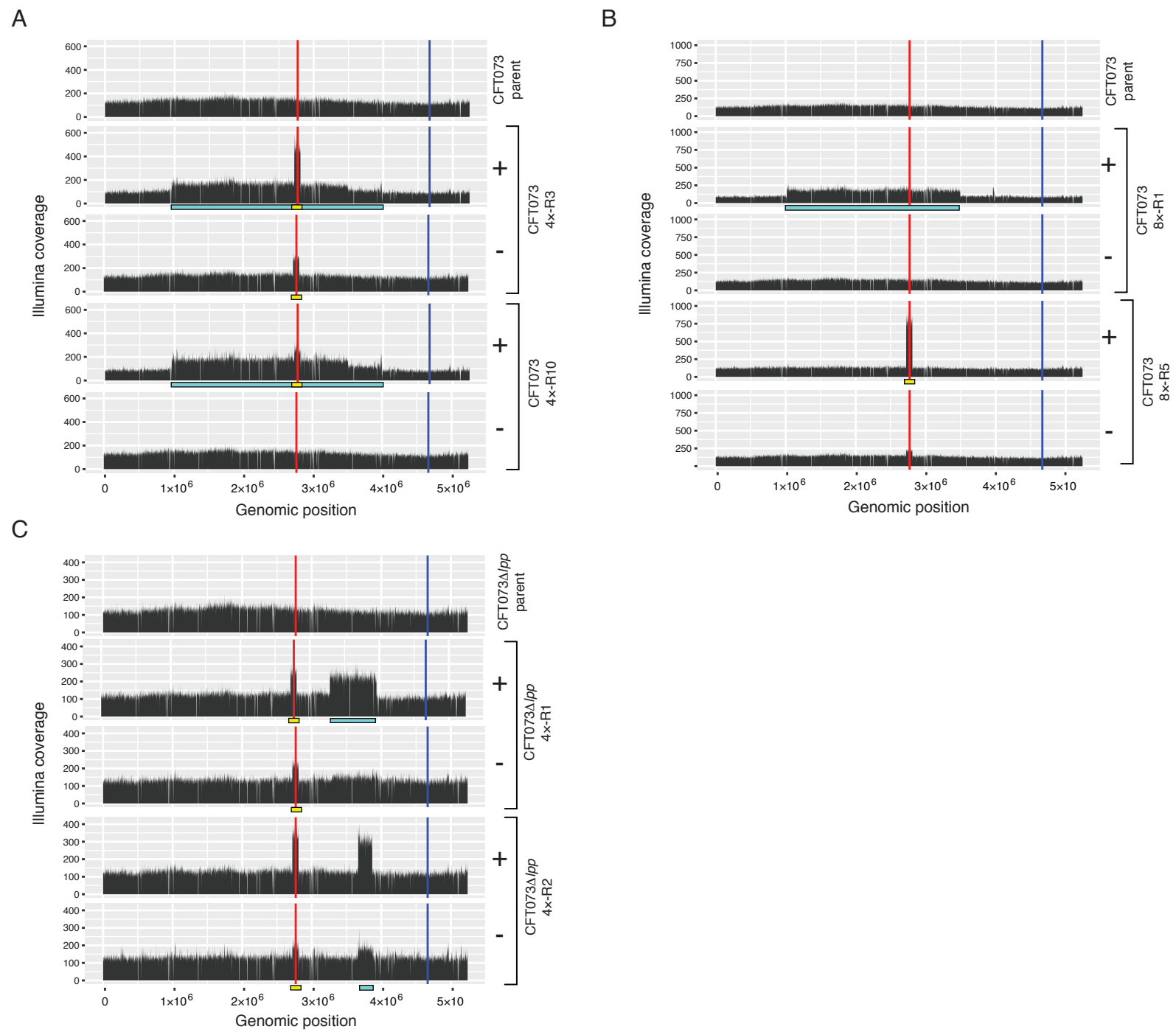

Figure S6

Supplement: FIG S6 [file mBio.02018-20-sf006.pdf]
