# Supplementary material for: Unstable Mechanisms of Resistance to Inhibitors of Escherichia coli Lipoprotein Signal Peptidase
Source: mBio. 2020 Sep 8;11(5):e02018-20. doi: 10.1128/mBio.02018-20 (PMC7482066; doi:10.1128/mBio.02018-20)
Supplement: TABLE S1 [file mBio.02018-20-st001.docx]

**Table S1:** Genomic amplifications in G0709 mutants that were subsequently subjected to passage as inferred using whole genome sequencing.

| **Strain** | **Genomic coordinates**  **for amplification (bp)** | **Amplification size (kb)** | **Amplification**  **point** | ***lspA* within amplified region** |
| --- | --- | --- | --- | --- |
| 4×-R3 | dup [yneL/hipA-flxA/  ydfW] (56 kb)  3456686-1245003  5221428-68233 | 3031  89 | *ISEc10-istB*  *IS200C* | Yes  Yes |
|  |  |  |  |  |
| 4×-R10 | 3456686-1245003  5221428-68233 | 3031  89 | *ISEc10-istB*  *IS200C* | Yes  Yes |
|  |  |  |  |  |
| 8×-R1 | 5221428-68233 | 89 | *IS200C* | Yes |
|  |  |  |  |  |
| 8×-R5 | ~3512599-1240150  5221428-68233 | 2949  89 | *yeeR-CL127*  *IS200C* | Yes  Yes |
|  |  |  |  |  |
| Δ*lpp* 4×-R1 | 5221428-68233  545269- 545269 | 89  672 | *IS200C*  *IS200C* | Yes  No |
|  |  |  |  |  |
| Δ*lpp* 4×-R2 | 5221428-68233  ~925386-~1127311 | 89  202 | *IS200C*  No detectable homology | Yes  No |
