# Supplementary material for: Unstable Mechanisms of Resistance to Inhibitors of Escherichia coli Lipoprotein Signal Peptidase
Source: mBio. 2020 Sep 8;11(5):e02018-20. doi: 10.1128/mBio.02018-20 (PMC7482066; doi:10.1128/mBio.02018-20)
Supplement: TABLE S2 [file mBio.02018-20-st002.doc]

**Table S2:** Bacterial strains and plasmids used in this study

| **Bacterial strains** | **Description** | **Reference** |
| --- | --- | --- |
| ***E. coli*** |  |  |
| MG1655 | *E. coli* K-12  F­- lambda-­ *ilvG­* negative, *rfb­-50 rph­-1* | ATCC 700926 |
| MG1655Δ*lspA* | MG1655 Δ*lspA*::kan with an arabinose-inducible integrated *lspA* copy | This study |
| MG1655Δ*lspA + pLMG18lspA* | MG1655 Δ*lspA*::kan with an arabinose-inducible integrated *lspA* copy containing the pLMG18 vector expressing *E.coli*/*P. aeruginosa*/*A. baumannii* *lspA* | This study |
| CFT073 | Bacteremia isolate, wild-type (O6:K2:H1) | ATCC 700928 |
| CFT073 pBAD*lspA* | CFT073 containing pBAD vector expressing *lspA* | This study |
| CFT073 pBAD | CFT073 containing an empty pBAD24 arabinose-inducible vector | This study |
| CFT073Δ*lpp* | CFT073 Δ*lgt*::kan containing an arabinose-inducible integrated *lgt* copy | (1) |
| CFT073Δ*lpp pBADlpp* | CFT073 Δ*lpp*::kan containing a pBAD24 vector expressing *lpp* | This study |
| CFT073*imp4213* | CFT073 carrying the *imp4213* allele in *lptD* | (1) |
| TOP10 | pWQ601, general cloning strain | Invitrogen |
| CFT073 pBLA-*gfp* | CFT073 containing pBLA vector expressing *gfp* | This study |
| *E. cloacae* 13047 | *E. cloacae* isolated from spinal fluid | ATCC |
| *K. pneumoniae* 700603 | *K. pneumoniae* isolated from urine of hospitalized patient | ATCC |
| *A. baumannii* 17978 | *A. baumannii* isolated from a 4-month old infant with fatal meningitis | ATCC |
| *S. aureus* USA300 | USA300 FPR3757 | Center for Staphylococcal Research, Nebraska |
| **Plasmids** |  |  |
| pKD4 | Kanamycin resistance (Kan^R^) cassette flanked by FRT (FLP recognition target) sites, oriRγ | (2) |
| pKD46 | Expresses the phage λ Red recombinase, Amp^R^, temperature sensitive, oriRγ | (3) |
| pLDR8 | Lambda integrase expression vector | ATCC 77357 |
| pLDR9 | Lambda att site integration vector | ATCC 77358 |
| pBAD24 | Arabinose inducible expression vector | ATCC 87399 |
| pBAD24-(*lspA/lpp*) | pBAD24 expressing LspA or Lpp | This study |
| pLMG18 | Low-copy-number IPTG-inducible expression vector with tetracycline marker | (4) |
| pLMG18-(*lspA^Ec^/lspA^Pa^/lsp^Ab^*) | pLMG18 expressing *lspA* from *E. coli*, *P. aeruginosa* or *A. baumanii* | This study |
| pBLA-*gfp* | pBLA vector expressing *gfp* | (4) |

**References**

1. **Diao J**, **Bouwman C**, **Yan D**, **Kang J**, **Katakam AK**, **Liu P**, **Pantua H**, **Abbas AR**, **Nickerson NN**, **Austin C**, **Reichelt M**, **Sandoval W**, **Xu M**, **Whitfield C**, **Kapadia SB**. 2017. Peptidoglycan Association of Murein Lipoprotein Is Required for KpsD-Dependent Group 2 Capsular Polysaccharide Expression and Serum Resistance in a Uropathogenic Escherichia coli Isolate. MBio, 2nd ed. **8**:e00603–17.

2. **Silhavy TJ**, **Kahne D**, **Walker S**. 2010. The bacterial cell envelope. Cold Spring Harb Perspect Biol **2**:a000414–a000414.

3. **Cowles CE**, **Li Y**, **Semmelhack MF**, **Cristea IM**, **Silhavy TJ**. 2011. The free and bound forms of Lpp occupy distinct subcellular locations in Escherichia coli. Mol Microbiol **79**:1168–1181.

4. **Storek KM**, **Auerbach MR**, **Shi H**, **Garcia NK**, **Sun D**, **Nickerson NN**, **Vij R**, **Lin Z**, **Chiang N**, **Schneider K**, **Wecksler AT**, **Skippington E**, **Nakamura G**, **Seshasayee D**, **Koerber JT**, **Payandeh J**, **Smith PA**, **Rutherford ST**. 2018. Monoclonal antibody targeting the β-barrel assembly machine of Escherichia coli is bactericidal. Proc Natl Acad Sci USA **115**:3692–3697.
