# Supplementary material for: Unstable Mechanisms of Resistance to Inhibitors of Escherichia coli Lipoprotein Signal Peptidase
Source: mBio. 2020 Sep 8;11(5):e02018-20. doi: 10.1128/mBio.02018-20 (PMC7482066; doi:10.1128/mBio.02018-20)
Supplement: TABLE S3 [file mBio.02018-20-st003.doc]

**Table S3:** Primers used in this study for strain generation and quantitative PCR

| **Primer** | **Sequence (5’ to 3’)** |
| --- | --- |
| ***Strain generation*** | |
| MG1655 Δ*lspA-*F | CGCTGTGTCAGCAACGTCGCCGGTGACGGTGAAAAACGTAAGTTTGCCTG AGTGTAGGCTGGAGCTGCTTC |
| MG1655 Δ*lspA-*R | CAATCTGTAGGCCGGATAAGATGCGTCAGCATCGCATCCGGCAGGGTTTA CATATGAATATCCTCCTTAGTTCCTATTC |
| ***Quantitative PCR*** | |
| *lspA-F* | TCAGCGCGTAAGCGATATT |
| *lspA-R* | GTTGGTTCTTTGCCGGTATTG |
| *LspA-PRB* | /56-FAM/ATGATGT ATCGCTCGAAGGCCACG/36-TAMSp/ |
| *lep B-F* | GATACCGCCAATGCGACTTA |
| *lepB-R* | TTGTGCCTGAAGCGAATCT |
| *lepB-PRB* | /56-FAM/ATAAGCAAGAAGGCGAATGGCCGA/36-TAMSp/ |
| *istB-F* | GCCAAGATCATCCACCACTATC |
| *istB-R* | CAGGAACTACGTAAAGCCAGAG |
| *istB-PRB* | /56-FAM/AGCTGCTACTGAAACTGGATCGCT /36-TAMSp/ |
| *ISEc10-F* | GCTGACAGAGAGCAGCATAA |
| *ISEc10-R* | GGAGTGCCGGT AGAACATAAA |
| *ISEc10-PRB* | /56-FAM/TGGAAACAACAGGGCGAAGATGGA/36-TAMSp/ |
| *lpp-F* | ACGAGCTGCGTCATCTTTAG |
| *lpp-R* | CTGTCTTCTGACGTTCAGACTC |
| *lpp-PRB* | /56-FAM/ACGCTAAAGTTGACCAGCTGAGCA/36-TAMSp/ |
| *rrsB-F* | TGCGATCTTCGGTGAGAAAG |
| *rrsB-R* | CATCGCGAGTAAAGACCTGAA |
| *rrsB-PRB* | /56-FAM/ACTCTTCTC/ZEN/TGCGCGTACCAAACG/31ABkFQ/ |
